# Physiologically Based Pharmacokinetic Modeling of Di(2-ethylhexyl) Adipate and Its Primary Metabolite, Mono(2-ethylhexyl) Adipate, in Rats, Incorporating Circulatory Topology-Based Multi-Tissue Metabolism and Lymphatic Absorption

**DOI:** 10.3390/pharmaceutics18081001

**Published:** 2026-08-13

**Authors:** Eunsuk Yang, Yoo-Seong Jeong, Minsang Kim, Seungchan Kim, Suk-Jae Chung

**Affiliations:** College of Pharmacy, Seoul National University, Seoul 08826, Republic of Korea; yes1342@snu.ac.kr (E.Y.); jus2401@snu.ac.kr (Y.-S.J.); minsang809@snu.ac.kr (M.K.); tmdcks2449@snu.ac.kr (S.K.)

**Keywords:** Di(2-ethylhexyl) adipate (DEHA), physiologically based pharmacokinetic (PBPK) modeling, extrahepatic metabolism, physiological circulatory topology, lymphatic transport

## Abstract

**Background/Objectives:** Di(2-ethylhexyl) adipate (DEHA), a biocompatible ester plasticizer, has gained interest as a potential pharmaceutical excipient, yet its pharmacokinetics remain poorly characterized. This study aimed to develop a physiologically based pharmacokinetic (PBPK) model for DEHA and its primary metabolite, mono(2-ethylhexyl) adipate (MEHA), in rats by integrating in vitro, in vivo, in silico, and physiological data. **Methods:** In vitro hydrolysis was evaluated across tissues using bis-(p-nitrophenyl) phosphate (BNPP) to distinguish BNPP-sensitive and -insensitive metabolism, and tissue-specific clearances were extrapolated to the whole body using a circulatory topology-based framework accounting for sequential extraction across tissues, venous blood, and lungs. **Results:** Both adipates underwent rapid BNPP-sensitive hydrolysis across multiple tissues, whereas DEHA additionally exhibited BNPP-insensitive metabolism. The framework yielded reasonable estimates of the observed arterial clearance in vivo. Following oral administration, DEHA showed a reproducible double-peak plasma profile, with lymphatic transport identified as the predominant absorption route responsible for the prolonged terminal phase. The final model adequately reproduced the plasma and mesenteric lymph concentration-time profiles of DEHA and MEHA following both intravenous and oral administration. **Conclusions:** By integrating multi-tissue metabolism, circulatory topology, and lymphatic absorption, this study provides a quantitative framework applicable to rapidly hydrolyzed, highly lipophilic ester compounds, including pharmaceutical excipients and ester prodrugs.

## 1. Introduction

Di(2-ethylhexyl) adipate (DEHA) is a lipid-like plasticizer widely used to improve the flexibility of polymeric products, including food-contact materials and medical devices. Originally developed as a safer alternative to di(2-ethylhexyl) phthalate (DEHP), DEHA now accounts for up to 2.5% of the global plasticizer market [1], with its consumption exceeding 24,000 tons in the United States and 14,000 tons in Western Europe as of 2014 [2].

Regulatory agencies have generally considered DEHA to be safe: based on a no-observed-effect level of 30 mg/kg/day identified in an animal teratogenicity study, the Scientific Committee on Food of the European Union has set a tolerable daily intake of 0.3 mg/kg/day [3] and a specific migration limit of 18 mg/kg for food-contact materials [4]. Similarly, the U.S. Food and Drug Administration (FDA) permits the use of DEHA as an indirect food additive [5]. Given its physicochemical properties, which are similar to those of biocompatible citrate-based plasticizers (e.g., acetyl tributyl citrate) already approved by the FDA for use as pharmaceutical coatings [6], DEHA has recently attracted attention as a potential pharmaceutical excipient [7]. DEHA has also been considered as a safer alternative to phthalates in the fabrication of PVC-based medical devices [8].

Despite its potential applicability in pharmaceutical formulations and medical devices, quantitative data on the systemic exposure and pharmacokinetic (PK) mechanisms of DEHA remain limited in the literature. A previous study [9] reported that, following oral administration of ^14^C-labeled DEHA in rats (500 mg/kg), nearly all radioactivity was eliminated within 48 h, primarily via urinary excretion (49.5%) and expired carbon dioxide (46.0%). Radioactivity was widely distributed to the major tissues, including the liver, kidneys, testes, and muscle, but declined substantially within 24 h post-dose [9]. Importantly, these measurements did not distinguish between the parent form and its metabolites, precluding a quantitative assessment of systemic exposure and the disposition of individual DEHA-related compounds.

Previously, a physiologically based pharmacokinetic (PBPK) model was proposed to describe the PK of DEHA and its metabolites in humans [10]. However, tissue distribution parameters were largely predicted in silico, metabolism was confined to the liver and gastrointestinal tract without accounting for the extrahepatic hydrolysis expected for these labile esters, and the model was calibrated against urinary metabolite data rather than measured plasma or tissue concentrations. Although a lymphatic compartment was included, its contribution was informed only indirectly by the urinary data, leaving the quantitative role of intestinal lymphatic transport uncertain. DEHA contains two ethylhexyl groups esterified to an adipate moiety, and esterases are widely distributed across mammalian tissues [11]. Therefore, DEHA and its primary metabolite, mono(2-ethylhexyl) adipate (MEHA), are anticipated to undergo rapid de-esterification in vivo, similar to the metabolic processes reported for DEHP [12]. Nevertheless, the tissue-specific disposition of DEHA and MEHA and the quantitative contributions of their metabolic pathways have not been systematically evaluated.

From a kinetic perspective, DEHA and MEHA present a unique opportunity to investigate the impact of extensive extrahepatic elimination on systemic drug disposition. When elimination occurs sequentially as the drug traverses multiple serially connected tissues before returning to arterial circulation, accurate predictions of arterial plasma clearance require explicit consideration of this extraction process. In addition, while intestinal lymphatic transport has been reported for highly lipophilic compounds [13], its quantitative contribution to systemic DEHA exposure remains unclear. Consequently, neither multi-tissue elimination nor lymphatic absorption has been explicitly incorporated into existing PK models of DEHA and MEHA.

The primary objective of this study was to develop a mechanistic PBPK model of DEHA and MEHA in rats that incorporates these kinetic features. Hydrolysis kinetics were systematically assessed in nine tissue homogenates to quantify the contribution of extrahepatic tissues to the overall clearance of DEHA and MEHA, thereby enabling an in vitro-in vivo extrapolation (IVIVE) workflow. In addition, DEHA concentrations were measured in mesenteric lymph following oral administration, allowing the observed double-peak phenomenon to be explicitly described through separate lymphatic and portal absorption pathways. The resulting model was evaluated with respect to DEHA and MEHA concentration-time profiles following both intravenous (IV) and oral administration in rats, demonstrating the importance of incorporating the relevant disposition mechanisms into PBPK models of rapidly hydrolyzed lipophilic esters.

## 2. Materials and Methods

### 2.1. Materials and Animals

Details of the materials, reagents, and the high-performance liquid chromatography-tandem mass spectrometry (HPLC-MS/MS) quantitation method used in this study are provided in the Supplementary Materials [14,15]. Male Sprague-Dawley (SD) rats (Koatech, Pyeongtaek, Republic of Korea) weighing 240–260 g were used for in vivo PK studies. For lymphatic fluid collection, larger rats weighing 300–330 g were used to facilitate visualization of the mesenteric lymphatic vessels. All animals were provided with standard chow and had free access to tap water. They were housed under a 12 h light/dark cycle in a temperature- and humidity-controlled environment at the Animal Center for Pharmaceutical Research of Seoul National University. Rats were fasted for 12 h before each experiment. All animal procedures were reviewed and approved by the Institutional Animal Care and Use Committee of Seoul National University.

### 2.2. Control of Analytical Contamination and Enzymatic Degradation

In preliminary studies, substantial DEHA contamination was detected in commonly used HPLC solvents, presumably due to prior contact with polymer-based materials. Therefore, deuterium-labeled DEHA (DEHA-d8) was used in experiments involving low DEHA concentrations. Assuming identical kinetic behavior, the administered doses and measured concentrations of DEHA-d8 (molecular weight: 378.6) were converted to DEHA-equivalent values (molecular weight: 370.6) and are reported as DEHA throughout this study. No comparable contamination was observed for MEHA.

Enzymatic hydrolysis of DEHA and MEHA was observed in rat plasma and tissue homogenates. Addition of phenylmethylsulfonyl fluoride [PMSF; 1 mM final concentration [16]], a serine protease/esterase inhibitor, prevented adipate degradation, and sample collection vials were therefore pretreated with PMSF when necessary to minimize post-collection degradation during the sample handling steps.

### 2.3. Systemic PK Study of DEHA and MEHA in Rats

Surgical and experimental procedures for the characterization of the PK of DEHA and MEHA in rats are described in the Supplementary Materials. Briefly, DEHA (5.94 mg/kg as DEHA equivalents), consisting of unlabeled (3 mg/kg) and deuterated (3 mg/kg) forms, or MEHA (5 mg/kg) was IV-administered to rats, and blood, urine, and bile samples were collected in separate studies to characterize their PK and excretion profiles. Urine and bile samples were divided into two aliquots: One was directly analyzed, while the other was treated with β-glucuronidase/sulfatase to quantify conjugated metabolites.

For oral administration studies, DEHA (99.8 mg/kg as DEHA equivalents), consisting of unlabeled (90 mg/kg) and deuterated (10 mg/kg) forms, was administered by oral gavage. To investigate intestinal lymphatic absorption, mesenteric lymph samples were collected following the surgical procedures described by Porter and Charman [17]. To minimize perturbation of intestinal absorption caused by surgery and lymph collection, only a single lymph sample was taken from each animal.

### 2.4. Metabolic Stability of DEHA and MEHA in Various In Vitro Incubation Systems

The metabolic stability of DEHA and MEHA was evaluated in rat liver microsomes, fasted-state simulated gastric fluid (FaSSGF), plasma, mesenteric lymph, homogenates prepared from nine rat tissues, and lipase reaction mixtures. Rat liver microsomes and FaSSGF were prepared according to the manufacturer’s instructions. Fresh plasma and mesenteric lymph were collected from rats for each experiment. Nine tissues (adipose, brain, gut, heart, liver, lung, kidney, muscle, and spleen) were collected and homogenized with three volumes of Dulbecco’s phosphate-buffered saline (DPBS; df=4) using an Ultra Turrax homogenizer (IKA, Staufen, Germany). Lipase reaction mixtures containing porcine pancreatic lipase (250 IU/mL) or cholesterol lipase (125 U/mL) were prepared in DPBS supplemented with 5 mM sodium taurocholate (pH 8.0). All reaction mixtures were preincubated at 37 °C for 5 min.

Incubations were initiated by adding DEHA or MEHA dissolved in dimethyl sulfoxide (DMSO) to achieve a final concentration of 0.6 µg/mL (final DMSO concentration ≤ 1%). All incubations were performed at 37 °C, and aliquots were collected at predetermined time points selected based on preliminary studies. To evaluate the involvement of carboxylesterase (CES), microsomes or tissue homogenates were pretreated with 1 mM bis-(p-nitrophenyl) phosphate (BNPP), a specific inhibitor of CES [18,19,20], for 10 min prior to substrate addition. The involvement of CYP enzymes was evaluated by measuring rat liver microsomal stability in the absence of an NADPH-regenerating system.

### 2.5. Unbound Fraction in Plasma ($f_{up}$) and Blood-to-Plasma Partition Coefficient (R) for DEHA and MEHA in Rats 

Due to the high lipophilicity and non-specific binding of DEHA, conventional rapid equilibrium dialysis (RED) was not suitable for determining its unbound fraction in rat plasma ($f_{up}$) [21]. Thus, the $f_{up}$ of DEHA was determined by means of solid-phase microextraction (SPME) according to [22], with minor modifications, whereas the $f_{up}$ of MEHA was determined using a RED device [21]. The blood-to-plasma partition coefficient (R) of DEHA and MEHA was experimentally determined. Detailed experimental procedures are described in the Supplementary Materials. The $f_{up}$ and R values were respectively calculated as the ratio of unbound to total plasma concentration ($f_{up}=C_{p,u}/C_{p}$) and that of the blood concentration to plasma concentration ($R=C_{B}/C_{p}$).

### 2.6. Parallel Artificial Membrane Permeability Assay (PAMPA)

The passive membrane permeability of DEHA and MEHA was evaluated using a parallel artificial membrane permeability assay (PAMPA) [23]. The assay was performed in a 96-well format consisting of donor and acceptor compartments filled with phosphate buffer (pH 7.4, 5% DMSO) and separated by an artificial membrane [1% (*w*/*v*) phosphatidylcholine in dodecane]. DEHA or MEHA (10,000 ng/mL) was added to the donor compartment and incubated for 16 h at room temperature. After incubation, compound concentrations in the donor and acceptor compartments were measured to calculate the apparent permeability coefficients [23]. Propranolol and mannitol were included as high- and low-permeability controls. To account for inter-laboratory variability, the measured permeability of propranolol was divided by a literature value (40 × 10^−6^ cm/s) [24], and the resulting ratio was applied to correct the permeability estimates of DEHA and MEHA.

### 2.7. In Vitro Estimation of the Tissue-to-Plasma Partition Coefficients ($K_{pT,pred}$) for PBPK Model Development of DEHA and MEHA in Rats

Because DEHA and MEHA underwent degradation in all tissues examined, the tissue-to-plasma ‘concentration ratios’ observed in vivo ($K_{pT,app}$) may differ from the tissue-to-plasma ‘partition coefficients’ ($K_{pT}$), which are independent of tissue elimination [25]. Accordingly, their in vitro predicted values ($K_{pT,pred}$) were obtained from the unbound fractions measured in homogenates of nine rat tissues under enzyme-inhibited conditions.

The protocol used to determine the unbound fraction in tissue homogenates ($f_{u,T,homogenate}$) was identical to that used to measure $f_{up}$ (see above). Both PMSF and BNPP (1 mM) were added to the homogenates to prevent degradation. The resulting $f_{u,T,homogenate}$ values were corrected for dilution using Equation (1) to obtain the unbound fraction in intact tissues ($f_{u,T}$) [26]. $K_{pT,pred}$ values were calculated using Equation (2) [27].

(1) $$f_{u,T}=\frac{1/df}{1/f_{u,T,homogenate}-1+1/df}$$

(2) $$K_{pT,pred}=\frac{f_{up}}{f_{u,T}}$$

For skin and bone, $K_{pT}$ values were estimated via in silico methods [28,29] with the corrected tissue-composition parameters reported in the corrigendum to Schmitt.

### 2.8. Extrapolation of In Vitro Stability Data to In Vivo Tissue Elimination Kinetics for DEHA and MEHA in Rats

In vitro degradation kinetics were scaled to estimate tissue-specific in vivo metabolic clearances of DEHA and MEHA. For hepatic clearance, the degradation kinetics measured in rat liver microsomes were extrapolated using a microsomal protein per gram of liver (MPPGL) value of 44.5 mg [30] and a liver weight of 8.57 g (LW; Simcyp V23 rat module, Certara UK Ltd., Sheffield, UK). The hepatic unbound intrinsic clearance ($CL_{u,LI,\;int}$) was calculated as follows:

(3) $$CL_{u,LI,int}(\mathrm{mL}/\min/\mathrm{rat})\;=\frac{\ln\left(2\right)\cdot MPPGL\cdot LW}{t_{1/2,invitro}\cdot f_{u,mic}\cdot {\left[P\right]}_{mic}}$$

where ${\left[P\right]}_{mic}$ is the microsomal protein concentration (0.5 mg/mL), $t_{1/2,in\;vitro}$ is the degradation half-life in the microsomal incubation mixtures, and $f_{u,mic}$ is the unbound fraction in the incubation system. Here, $f_{u,mic}$ was assumed to be identical to the $f_{up}$ [31].

For extrahepatic tissues, the disappearance rate constant measured in tissue homogenates ($k_{T,homogenate}$) was corrected for dilution and non-specific binding to obtain the unbound disappearance rate constant ($k_{u,T,total}$) (Equation (4)).

(4) $$k_{u,T,total}=k_{T,homogenate}\cdot df/f_{u,T,homogenate}$$

DEHA hydrolysis in tissue homogenates was only partially inhibited by BNPP, indicating the presence of both CES-mediated and BNPP-insensitive elimination pathways. Given that circulating lipids undergo intravascular lipolysis mediated by vascular lipases prior to cellular uptake [32], the unbound disappearance rate ($k_{u,T,total}$) was partitioned into the tissue parenchymal (${CL}_{u,T,int}$) and microvascular (${CL}_{u,T,vas,int}$) components according to the BNPP-inhibited fraction ($f_{BNPP}$):

(5) $${CL}_{u,T,int}=k_{u,T,total}\cdot V_{T}\cdot f_{BNPP}$$

(6) $${CL}_{u,T,vas,int}=k_{u,T,total}\cdot V_{T}\cdot {(1-f}_{BNPP})$$

In contrast, MEHA instability was almost completely inhibited by BNPP and was thus assumed to occur exclusively via the ${CL}_{u,T,int}$ pathway ($f_{BNPP}=1$).

Based on these parameters, the tissue elimination of DEHA and MEHA was incorporated into an extended clearance model assuming symmetric transport [33]. Accordingly, the tissue or organ clearance ($CL_{T}$) was expressed as

(7) $$CL_{T}=\frac{Q_{T}\cdot f_{d}\cdot f_{up}\cdot CL_{u,T,int}}{Q_{T}\cdot f_{d}\cdot R+f_{up}\cdot CL_{u,T,int}}$$

where $f_{d}$ is the fractional distribution parameter, defined as the fraction of drug delivered by the tissue blood flow that effectively permeates into the tissue [33]. Of note, the term $CL_{T}$ is defined as acting upon the inlet concentration, i.e., arterial plasma concentration for most tissues, whereas the hepatic and pulmonary clearances were referenced to the corresponding physiological inlet plasma concentrations (see the Supplementary Materials). The microvascular clearance ($CL_{T,vas}$) was defined with respect to the arterial plasma (for most tissues) or venous plasma (for the lung), expressed as:

(8) $$CL_{T,vas}={CL}_{u,T,vas,int}\cdot f_{up}$$

In addition, the clearances directly occurring within the arterial ($CL_{p,art}$) and venous ($CL_{p,ven}$) plasma matrices were defined as:

(9) $$CL_{p,art}=k_{plasma,invitro}\cdot V_{p,art}$$

(10) $$CL_{p,ven}=k_{plasma,invitro}\cdot V_{p,ven}$$

where $k_{plasma,invitro}$ is the disappearance rate constant measured in plasma, and $V_{p,art}$ and $V_{p,ven}$ are the arterial and venous plasma volumes.

### 2.9. Calculation of Systemic Clearance Incorporating Circulatory Topology-Based Multi-Tissue Metabolism

**Figure 1:**
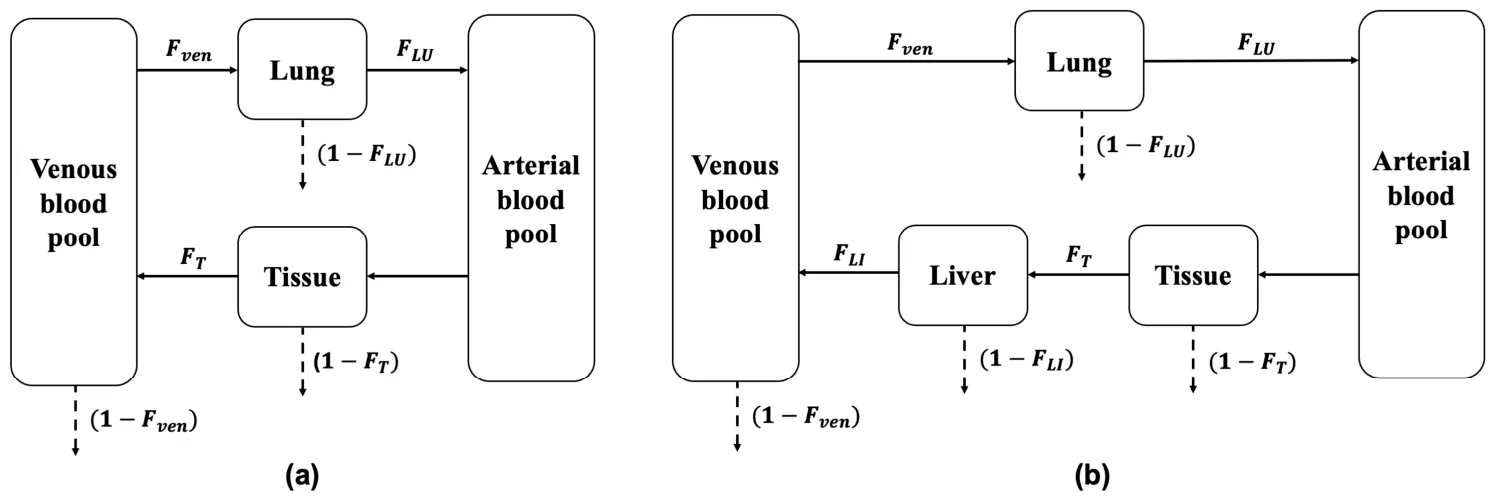
Schematic representation of the circulatory topology with serially connected eliminating organs. (**a**) Non-splanchnic circulation. (**b**) Splanchnic circulation. Solid and dashed arrows indicate blood flow and elimination pathways. $F_{X}$ denotes the fraction transferred from a given compartment to the next compartment ($F_{ven}$, venous return; $F_{LU}$, lung; $F_{T}$, tissue; $F_{LI}$, liver), and (1 − $F_{X}$) denotes the corresponding eliminated fraction. The amount reaching each compartment equals the initial input multiplied by the product of the preceding fractions.

As described above, tissue-specific clearance components ($CL_{T}$ and $CL_{T,vas}$) were estimated from in vitro data. Because tissues responsible for elimination are arranged in series within the circulatory system, the observed systemic clearance with respect to the arterial plasma cannot be represented by a simple sum of individual tissue clearances. This applies to both the general circulation (tissues-venous blood-lung; Figure 1a) and the splanchnic circulation (splanchnic tissues-liver-venous blood-lung; Figure 1b), where sequential extraction occurs along the circulatory pathway.

To account for this topology-dependent sequential extraction, the arterial plasma clearance was evaluated at the level of each individual circulatory pathway ($CL_{path}$) rather than isolated tissues. The total systemic clearance ($CL_{sum}$) was defined as the sum of pathway-specific clearances ($\sum CL_{path}$), together with metabolic contributions occurring directly within the arterial vasculature ($\sum CL_{T,vas,art}$) and arterial plasma ($CL_{p,art}$):

(11) $$CL_{sum}=\sum CL_{path}+\sum CL_{T,vas,art}+CL_{p,art}$$

For each pathway, the $CL_{path}$ was calculated as the product of the pathway blood flow ($Q_{path}$), the R, and the overall extraction ratio along the pathway ($ER_{path}$):

(12) $$CL_{path}=\;Q_{path}\cdot R\cdot ER_{path}$$

The $ER_{path}$ was calculated according to the corresponding venous drainage topology. For non-splanchnic tissues and the liver (Figure 1a), sequential extraction occurred through the tissue, venous blood, and the lungs. For splanchnic tissues (gut and spleen) (Figure 1b), blood additionally passed through the liver before entering the venous circulation. The availability of each compartment is denoted as F, and $ER_{path}$ was calculated as one minus the product of the availability values along the corresponding pathway:

(13) $$\mathrm{Non}\text{-}\mathrm{splanchnic}\;\mathrm{tissues}\;\mathrm{and}\;\mathrm{liver}:\;{ER}_{path}=1-\left(F_{T}\cdot F_{ven}\cdot F_{LU}\right)$$

(14) $$\mathrm{Gut}\;\mathrm{and}\;\mathrm{spleen}:\;{ER}_{path}=1-\left(F_{T}\cdot {F_{LI}\cdot F_{ven}\cdot F}_{LU}\right)$$

(15) $$\mathrm{Rest}\;\mathrm{of}\;\mathrm{the}\;\mathrm{body}:\;{ER}_{path}=1-\left({F_{ven}\cdot F}_{LU}\right)$$

In these equations, T, LI, LU, and ven denote the tissue of interest, the liver, the lungs, and the venous compartment, respectively. Under the assumption of linear PK, individual pathways were treated independently when calculating $ER_{path}$.

Assuming linear elimination and permeability-limited distribution [33], $F_{T}$ and $F_{ven}$ were calculated from their respective extraction kinetics (Equations (16) and (17)):

(16) $$F_{T}=1-\frac{f_{d}\cdot f_{up}\cdot CL_{u,T,int}}{Q_{T}\cdot f_{d}\cdot R+f_{up}\cdot CL_{u,T,int}}$$

(17) $$F_{ven}=\frac{Q_{CO}\cdot R}{Q_{CO}\cdot R+CL_{p,ven}+CL_{T,vas,LU}}$$

### 2.10. Non-Compartmental Analysis of Plasma PK Data for DEHA and MEHA

The standard non-compartmental analysis (NCA) was applied to the arterial plasma concentration-time data following IV or oral administration of DEHA and MEHA [27]. Consistent with the circulatory topology described above, IV-administered DEHA or MEHA undergoes extraction in the venous blood and the lungs before reaching the arterial circulation. Therefore, the effective dose available to the arterial circulation ($Dose_{eff}$) was calculated as:

(18) $$Dose_{eff}=\;Dose\cdot (F_{ven}\cdot F_{LU})\;$$

which was subsequently used in place of the administered dose for the calculation of NCA parameters, including the mean residence time (MRT), the total plasma clearance (CL or CL/F) and the steady-state volume of distribution ($V_{SS}$).

### 2.11. Determination of the Fraction of the Administered DEHA Dose Converted to MEHA ($f_{m}$)

To determine the fraction of the administered DEHA dose converted to MEHA in vivo ($f_{m}$), DEHA [unlabeled (3 mg/kg) and deuterated (3 mg/kg); see above] was IV-administered to rats. The resulting plasma PK profiles of DEHA and MEHA were analyzed assuming linear and stationary kinetics. Here, $f_{m}$ was calculated as:

(19) $$f_{m}=\frac{AUC_{MEHA}\cdot CL_{MEHA}/MW_{MEHA}}{{Dose}_{DEHA}/MW_{DEHA}}$$

where ${AUC}_{MEHA}$ is the AUC of MEHA following DEHA administration, $CL_{MEHA}$ denotes the systemic clearance of MEHA, $MW_{MEHA}$ and $MW_{DEHA}$ are the molecular weights of MEHA and DEHA, and ${Dose}_{DEHA}$ represents the administered DEHA dose. For comparison, ${CL}_{MEHA}$ was assumed to be either $CL_{sum}$ or CL.

### 2.12. PBPK Modeling for DEHA and MEHA in Rats

**Figure 2:**
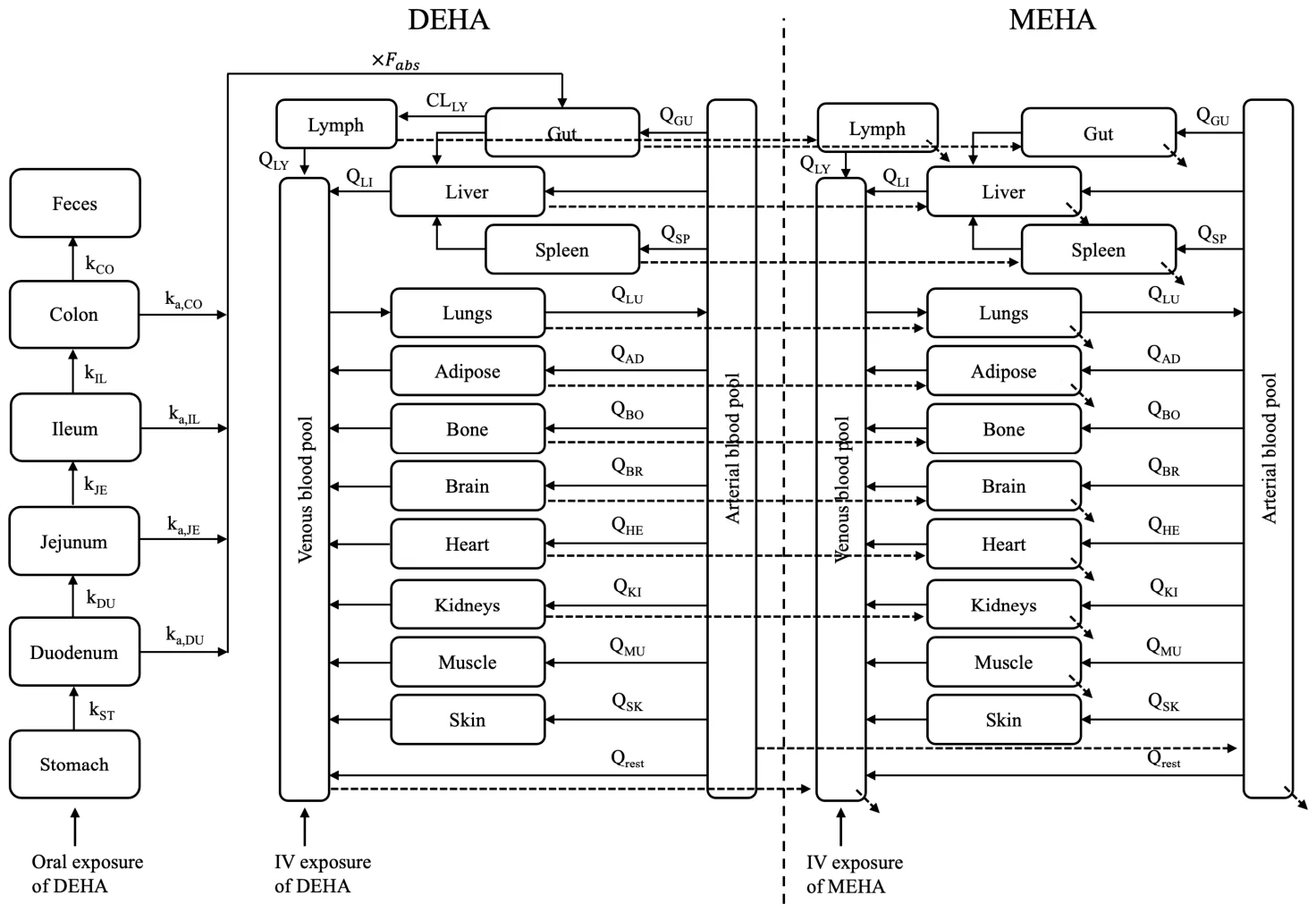
Schematic representation of the PBPK model for DEHA and MEHA in rats. The model consists of eleven tissue compartments together with arterial and venous blood pools and an additional gastrointestinal-lymphatic module describing oral DEHA absorption. Dashed arrows indicate elimination pathways, and DEHA hydrolysis represents the formation of MEHA. Symbols are defined in the Supplementary Materials.

A PBPK model was developed to describe the plasma PK profiles of DEHA and MEHA in rats following IV and oral administration (Figure 2). The complete model equations are provided in the Supplementary Materials, and compound-specific parameter values are summarized in Section 3. When necessary, physicochemical properties were obtained using Chemicalize (ChemAxon Ltd., Budapest, Hungary). Physiological parameters for a 250 g rat were obtained from the Simcyp V23 rat module (Certara UK Ltd., Sheffield, UK) and are listed in Supplementary Tables S1 and S2 [34].

The whole-body model consisted of eleven tissue compartments, arterial and venous blood pools, along with an additional lymph compartment incorporated for both adipates. The distribution kinetics were described using the permeability-limited model [33], and the tissue-specific clearance terms ($CL_{T}$ and $CL_{T,vas}$) were assigned to the corresponding tissues. The conversion of DEHA to MEHA was assumed to be complete in the model.

To describe oral DEHA absorption, a gastrointestinal (GI)-lymphatic PBPK model adapted from Tao et al. [34] was incorporated. The model accounted for Weibull dissolution, GI transit, and region-dependent absorption, wherein DEHA absorbed into the gut is subjected to lymphatic transfer ($CL_{LY}$), gut elimination, and portal-hepatic transfer. DEHA entering the lymphatic system was assumed to reach the venous circulation via the thoracic duct after a lymphatic lag time ($T_{lag,LY}$). The lymph flow rate ($Q_{LY}$) was fixed at 2.5 mL/h [35] and the lymph volume was assumed to be equal to the blood volume [36]. Because oral DEHA administration resulted in extremely high DEHA lymph concentrations, the hydrolysis of DEHA to MEHA in mesenteric lymph was modeled as a saturable hydrolysis process based on the Michaelis-Menten kinetics ($K_{m,LY}$).

Parameter estimation was performed using the maximum likelihood method in ADAPT 5 (Biomedical Simulations Resource, University of Southern California, Los Angeles, CA, USA) [37]. For the disposition kinetics of DEHA, membrane permeability ($P_{PAMPA,DEHA}$), which could not be reliably determined experimentally, and the $K_{p,scalar}$ value of DEHA were estimated by simultaneously fitting the plasma DEHA data following IV DEHA administration and plasma MEHA data formed after IV DEHA administration. For the oral absorption kinetics of DEHA, the $CL_{LY}$, $T_{lag,\;LY}$, $K_{m,LY}$, and the absorbed fraction ($F_{abs}$) were estimated from the plasma and mesenteric lymph concentration-time data following oral DEHA administration, with the disposition parameters fixed. The precision of each estimate was expressed as its coefficient of variation, and the parameter correlation matrices from ADAPT 5 were examined to assess identifiability (Supplementary Table S4). The corresponding maximal hydrolysis velocity ($V_{max,LY}$) was not estimated independently but was constrained as $V_{max,LY}=k_{LY,\;in\;vitro}\cdot K_{m,LY}$, where $k_{LY,\;in\;vitro}$ is the first-order disappearance rate constant determined in vitro. No model fitting was conducted for MEHA PK parameters, enabling forward model simulation of the plasma MEHA profiles following IV MEHA administration.

### 2.13. Statistical Analysis and Model Performance Assessment

Experimental data are presented as the mean ± standard deviation unless otherwise specified. When appropriate, differences between groups were assessed using Student’s *t*-test. Because each group included only three replicates, normality was not formally assessed, and Student’s t-test was applied under the assumption of approximate normality. A *p*-value of < 0.05 was considered statistically significant.

The PBPK models were numerically integrated using the fourth-order Runge–Kutta method implemented in Berkeley Madonna (version 10.6.1; Berkeley Madonna, Inc., Albany, CA, USA). GraphPad Prism (version 10.0.0; GraphPad Software, San Diego, CA, USA) was used for data visualization. Model performance was evaluated using the mean squared log difference (MSLD) and the absolute average fold error (AAFE), calculated as:

(20) MSLD=∑logCpred−logCobs2N

(21) $$AAFE=10^{\sum \left|log(C_{pred}/C_{obs})\right|/N}$$

where $C_{obs}$ and $C_{pred}$ are the observed and predicted concentrations, and N is the number of observations.

## 3. Results

### 3.1. Plasma Protein Binding, Blood Partitioning, and Membrane Permeability of DEHA and MEHA

The $f_{up}$ values were determined to be 0.0208 ± 0.00220 for DEHA and 0.0278 ± 0.00483 for MEHA in rats. The R values were 0.538 ± 0.0137 for DEHA and 0.592 ± 0.0321 for MEHA in rats, indicating that neither adipate was extensively associated with blood cells. Because the theoretical lower limit of R is constrained by the plasma volume fraction, the R value for DEHA was fixed at 0.581 (1—hematocrit; Supplementary Table S2) in the model.

The PAMPA permeability of MEHA ($P_{PAMPA,MEHA}$) was 48.8 ± 4.26 × 10^−6^ cm/s, with a recovery of 90.3 ± 1.44%. In contrast, DEHA showed negligible recovery in the donor and acceptor compartments (2.33 ± 1.41%), suggesting that highly lipophilic DEHA partitioned into and was retained within the phospholipid bilayer rather than permeating across the artificial membrane. Consequently, reliable estimation of the PAMPA permeability of DEHA ($P_{PAMPA,DEHA}$) was not feasible, and a model-dependent value was instead estimated by means of PBPK model fitting (see below).

### 3.2. In Vitro Metabolic Stability of DEHA and MEHA in Various Incubation Systems

Table 1 summarizes the disappearance rate constants (k), half-lives ($t_{1/2}$), and the effects of BNPP across the biological matrices examined. Both DEHA and MEHA were generally unstable ($t_{1/2}$ = 0.08–32.2 min), except in urine, bile, and FaSSGF. BNPP markedly slowed MEHA degradation to varying extents across most matrices. Notably, DEHA degraded rapidly in pancreatic lipase ($t_{1/2}$ = 3.09 min) and cholesterol lipase ($t_{1/2}$ = 2.42 min) reaction mixtures, while MEHA remained stable under these conditions.

Exclusion of NADPH from liver microsomal incubations did not alter the disappearance rates of either DEHA (0.0892 ± 0.0031 vs. 0.0928 ± 0.0117 min^−1^) or MEHA (0.811 ± 0.0690 vs. 0.792 ± 0.0713 min^−1^), suggesting negligible involvement of CYP enzymes in their metabolism. 

### 3.3. Tissue Distribution of DEHA and MEHA Using Tissue and Plasma Unbound Fractions

Table 2 summarizes the $f_{u,T,homogenate}$, $f_{u,T}$, and $K_{pT,pred}$ values for DEHA and MEHA across nine rat tissues. For DEHA, $f_{u,T,homogenate}$ ranged from 0.0106 ± 0.00148 in the lung to 0.164 ± 0.0225 in the gut, corresponding to $f_{u,T}$ values of 0.00268–0.0469. Accordingly, $K_{pT,pred}$ was highest in the lung (7.76) and lowest in the gut (0.444). For MEHA, $f_{u,T,homogenate}$ ranged from 0.131 ± 0.0131 (lung) to 0.684 ± 0.121 (kidney), and $f_{u,T}$ ranged from 0.0362 to 0.351. Accordingly, $K_{pT,pred}$ ranged from 0.166 (muscle) to 0.769 (lung).

### 3.4. In Vivo PK Characterization of DEHA and MEHA Following IV and Oral Administration in Rats

**Figure 3:**
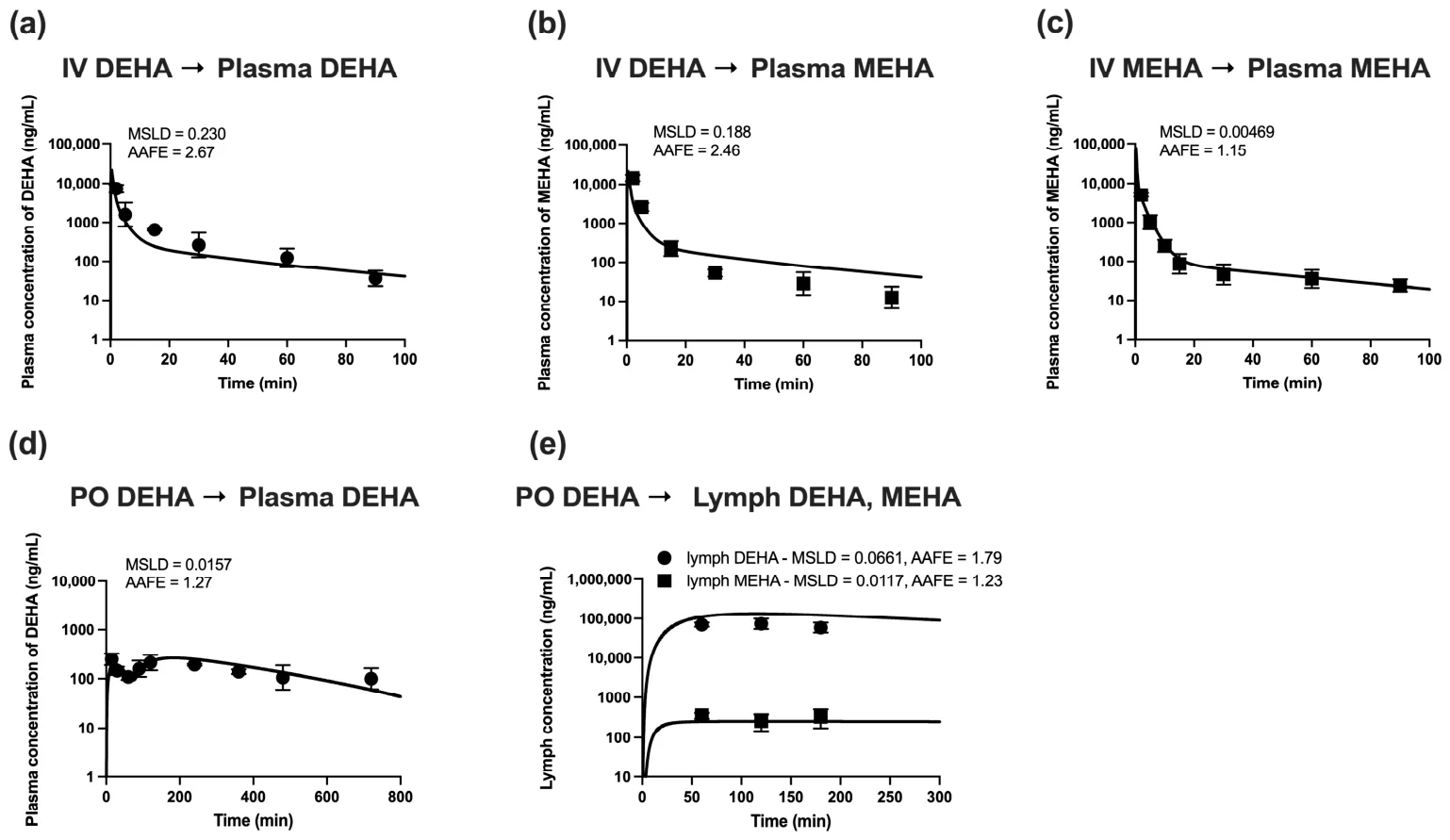
Plasma and mesenteric lymph concentration-time profiles of DEHA and MEHA following IV and oral administration in rats. (**a**) Plasma DEHA and (**b**) plasma MEHA following IV administration of DEHA (5.94 mg/kg). (**c**) Plasma MEHA following IV administration of MEHA (5 mg/kg). (**d**) Plasma DEHA and (**e**) mesenteric lymph DEHA and MEHA following oral administration of DEHA (99.8 mg/kg). Circles and squares in panel (**e**) denote DEHA and MEHA. Symbols denote observed data (mean ± standard deviation) and solid lines denote model fits for panels (**a**,**b**,**d**,**e**), and a model simulation for panel (**c**). MSLD and AAFE values are shown in each panel.

The plasma concentration-time profiles following the IV administration of DEHA (5.94 mg/kg) and MEHA (5 mg/kg) and the oral administration of DEHA (99.8 mg/kg) are shown in Figure 3. PK parameters calculated from NCA are summarized in Table 3. Following IV administration, the CL values of DEHA (84.2 ± 19.7 mL/min/kg) and MEHA (120 ± 10.6 mL/min/kg) were comparable to or greater than the hepatic blood flow in rats (77.4 mL/min/kg), consistent with their substantial extrahepatic elimination. The $V_{ss}$ values were 958 ± 116 mL/kg for DEHA and 1348 ± 866 mL/kg for MEHA. The plasma concentrations of both adipates declined in a multi-exponential manner, with the terminal half-lives ($t_{1/2}$) of 20.0 ± 5.83 min for DEHA and 34.7 ± 24.4 min for MEHA.

The $t_{1/2}$ value of MEHA found after IV DEHA administration (35.1 ± 22.7 min) was comparable to that observed after the IV administration of MEHA. Using Equation (19), the in vivo $f_{m}$ was estimated to be 176 ± 29.4% when $CL_{MEHA}$ was set equal to CL; this outcome was 119 ± 20.0% when $CL_{MEHA}$ was set equal to $CL_{sum}$ (see below).

Following oral DEHA administration, the bioavailability (F) of DEHA was 17.0 ± 8.43% (Table 3), whereas MEHA was not detected in plasma (<3 ng/mL). Compared to IV administration, oral DEHA exhibited a markedly prolonged $t_{1/2}$ value (563 ± 181 min), accompanied by a double-peak plasma concentration-time profile (Figure 3d). Given the high lipophilicity of DEHA (clog P = 6.83), mesenteric lymph concentrations were subsequently determined following oral administration (Figure 3e). Despite the limited number of observations, the apparent $t_{1/2}$ in lymph (556 min) was comparable to that in plasma, consistent with prolonged lymphatic transport contributing to systemic absorption.

Regarding excretory pathways, neither DEHA nor MEHA was detected (<3 ng/mL) in bile samples collected up to 180 min post-dose, regardless of the β-glucuronidase treatment, indicating negligible biliary excretion of the parent compounds or their glucuronide conjugates. Similarly, urinary recovery over 24 h ($f_{e}$) accounted for less than 0.01% of the administered dose for both adipates. Collectively, renal and biliary excretion contributed negligibly to the overall elimination of DEHA and MEHA.

### 3.5. PBPK Modeling for DEHA and MEHA PK in Rats

A PBPK model for DEHA and MEHA was developed by integrating in vivo, in vitro, and in silico datasets. Compound-specific (Table 4) and system-specific (Tables S1 and S2) input parameters were applied in PBPK equations described in the Supplementary Materials. Briefly, the tissue-specific elimination (Table 1) and binding (Table 2) parameters were used to calculate $CL_{u,T,int}$ (Equation (5)) and ${CL}_{u,T,vas,int}$ (Equation (6)). The homogenate-derived ${\;K}_{pT,pred}$ values (Table 2) were used to calculate the PBPK model-applicable $K_{pT}$ values. The final PBPK model calculations are presented in Figure 3, along with the observed data.

For MEHA, no further adjustment was required to reproduce the observed plasma concentration-time profile, as depicted in Figure 3c (i.e., forward simulation). The apparent permeability coefficient of MEHA ($f_{up}\cdot P_{PAMPA,MEHA}/R$) was calculated and found to be 2.29 × 10^−6^ cm/s, indicating that the distribution kinetics of MEHA in tissues essentially follow a perfusion-limited distribution [38].

For DEHA, the two distribution kinetic parameters, i.e., $P_{PAMPA,DEHA}$ and $K_{p,scalar}$, were simultaneously fitted to the plasma PK data for DEHA and MEHA following IV administration of DEHA (Figure 3a,b). The $P_{PAMPA,DEHA}$ estimate of 0.805 × 10^−6^ cm/s with reasonable precision (CV% = 36.1%) indicates that the DEHA distribution is permeability-limited (i.e., $f_{up}{\cdot P}_{PAMPA,DEHA}/R$ = 0.0288 × 10^−6^ cm/s) [38]. Based on the relationship $K_{pT}=K_{pT,pred}\cdot K_{p,scalar}$ applied to the nine rat tissues, the estimated $K_{p,scalar}$ was 0.542 (CV% = 60.0%), indicating that the in vitro-based $K_{pT,pred}$ values provided a reasonable basis for describing the tissue distribution kinetics of DEHA.

For the oral PK of DEHA, the four absorption kinetic parameters, i.e., $F_{abs}$, $CL_{LY}$, $K_{m,LY},$ and $T_{lag,LY}$, were fitted to the PK data in plasma (Figure 3d) and mesenteric lymph (Figure 3e). The estimated $F_{abs}$ value of 0.139 (CV% = 10.2%) was comparable to the observed F (0.170). The estimated lymphatic absorption parameters, $CL_{LY}$ (33.8 mL/min, CV% = 21.4%) and $T_{lag,LY}$ (66.5 min, CV% = 14.2%), adequately reproduced the double-peak phenomenon observed in plasma (Figure 3d). The estimated $K_{m,LY}$ value of 4640 ng/mL (CV% = 19.3%) characterized the saturable hydrolysis of DEHA within the lymphatic compartment and enabled the model to simultaneously describe the mesenteric lymph concentrations of both DEHA and MEHA following oral DEHA administration (Figure 3e). The estimated parameters showed acceptable precision and generally limited correlations. Pairwise correlations among the four oral parameters were low (|*r*| ≤ 0.29), whereas $P_{PAMPA,DEHA}$ and $K_{p,scalar}$ showed a correlation of *r* = 0.75 (Supplementary Table S4). The resulting model indicated that the lymphatic pathway was the predominant route of DEHA absorption (90.0%), with only a minor contribution from the portal-hepatic route (10.0%).

Interestingly, the model well predicted the mesenteric lymph DEHA concentration measured at 240 min after IV DEHA administration ($C_{obs}$ = 231 ± 104 ng/mL vs. $C_{pred}$ = 211 ng/mL), even though these data were not used during parameter estimation. While MEHA was experimentally not detectable in plasma following oral DEHA administration (<3 ng/mL; see above), the model predicted plasma MEHA concentrations ranging from 59.4 ng/mL to 157 ng/mL; however, such predictions remain experimentally unverified.

The model performance is summarized in Table S3. The current model adequately reproduced the exposure metrics ($C_{max}$ and AUC) for most of the datasets, with some deviations noted for early-time data. In terms of MSLD and AAFE parameters, the current model produced values less than 0.230 (MSLD) and 2.67 (AAFE) for all datasets in Figure 3, indicative of reasonable model performance.

### 3.6. Calculation of Clearance Values for DEHA and MEHA Based on the Circulatory Topology

The circulatory topology-based framework reconstructed systemic clearance with respect to arterial plasma ($CL_{sum}$) from bottom-up tissue clearance components (Table 5). $CL_{sum}$ was obtained as the sum of the pathway-specific organ clearances ($\sum CL_{path}$), the arterial vascular (lipase-mediated) clearances ($\sum CL_{T,vas,art}$), and the arterial plasma clearance ($CL_{p,art}$). Of note, lymphatic elimination was not included in the calculation given that lymph flow contributes only minimally to arterial circulation (0.0521% of the cardiac output; Supplementary Table S1).

For DEHA, the reconstructed $CL_{sum}$ was 81.3 mL/min/kg, which was comparable to the NCA-derived CL (84.2 ± 19.7 mL/min/kg). The arterial vascular clearance term ($\sum CL_{T,vas,art}$, 57.8 mL/min/kg) accounted for approximately 71.1% of $CL_{sum}$, suggesting that arterial vascular elimination may be a major contributor to systemic DEHA clearance. For MEHA, the reconstructed $CL_{sum}$ (81.7 mL/min/kg) was lower than the corresponding NCA-derived CL (120 ± 10.6 mL/min/kg). Nevertheless, the PBPK model adequately reproduced the observed plasma concentration-time profiles of both DEHA and MEHA (Figure 3a,c; Supplementary Table S3). Therefore, the bottom-up clearance estimates summarized in Table 5 were retained without empirical adjustment in the final PBPK model (see above).

## 4. Discussion

In this study, we developed a PBPK model for the quantitative characterization of the systemic PK of DEHA and its primary metabolite, MEHA, in rats. The establishment of a bottom-up in vitro-in vivo extrapolation (IVIVE) workflow is a crucial step in PBPK modeling, not only for mechanistically understanding in vivo kinetics but also for enabling future extrapolation across compounds and species using experimentally measurable parameters. To the best of our knowledge, this study provides the first experimentally informed PBPK framework for these rapidly hydrolyzed adipates by explicitly incorporating multi-tissue metabolism, circulatory topology, and lymphatic absorption into a unified model (Figure 2).

The circulatory topology-based approach enabled quantitative translation of tissue-specific in vitro metabolic clearances to the whole body by accounting for sequential extraction along eliminating tissues, venous blood, and lungs (Figure 1). In addition, the prolonged systemic disposition of highly lipophilic DEHA following oral administration was described by incorporating parallel portal and lymphatic absorption pathways, with lymphatic transport identified as the predominant route of its systemic absorption. Despite these complexities, which are difficult to address using conventional PK analysis, the model adequately reproduced the observed plasma and mesenteric lymph PK profiles of both DEHA and MEHA (Figure 3).

A major challenge during the development of the PBPK model was the estimation of tissue-specific distribution and elimination kinetic parameters. Because DEHA and MEHA contain labile ester linkages, both adipates underwent rapid hydrolysis in rat plasma and multiple tissue homogenates (Table 1). Consequently, the apparent tissue-to-plasma concentration ratios measured in vivo ($K_{pT,app}$) do not represent the true partition coefficients because tissue elimination occurs concurrently with the tissue distribution [25]. To overcome this limitation, tissue homogenates were incubated under enzyme-inhibited conditions to separately characterize tissue binding ($f_{u,T,homogenate}$) and metabolic instability ($k_{T,homogenate}$). This strategy provided a foundation for parameterizing both the tissue distribution and elimination within the PBPK model.

Extensive extrahepatic metabolism was incorporated through bottom-up scaling of the in vitro stability data. Inhibition studies identified CES as the primary enzyme responsible for MEHA hydrolysis, whereas the incomplete inhibition of DEHA degradation by BNPP indicated the involvement of additional BNPP-insensitive pathways, potentially including vascular lipases. Because DEHA rapidly disappeared in lipase-containing incubation systems (Table 1), vascular lipase-mediated clearance was incorporated into the PBPK model (Equation (8)). This process proved essential for reproducing the rapid early formation of MEHA following IV DEHA administration (Figure 3b), suggesting the importance of accounting for vascular lipase-mediated metabolism of highly lipophilic ester compounds. In contrast to a previous PBPK model of DEHA, which assumed that MEHA formation occurred only in the liver and gastrointestinal tract [10], our IVIVE analysis suggested that the vascular compartment may substantially contribute to DEHA hydrolysis. Under this interpretation, the estimated $\sum CL_{T,vas,art}$ accounted for approximately 71% of the systemic CL (Table 5). However, this finding should be regarded as a model-based hypothesis supported by available indirect evidence rather than a directly demonstrated metabolic process.

A key conceptual advance of this study was the reconstruction of systemic clearance within a physiological circulatory topology. Unlike conventional parallel summation, this approach accounts for sequential extraction across serially connected eliminating tissues. A sensitivity analysis (Supplementary Figure S1) demonstrated that neglecting this topology can substantially bias systemic clearance estimates, particularly when pulmonary extraction is significant.

The impact of this approach was illustrated by estimating the fraction of DEHA converted to MEHA ($f_{m}$). Because hydrolysis of either ester bond in DEHA yields MEHA, $f_{m}$ is expected to be close to, but not to exceed, unity. However, using the NCA-derived MEHA clearance (120 mL/min/kg) yielded a physiologically implausible $f_{m}$ value of 176 ± 29.4%. This overestimation highlights an inherent limitation of conventional NCA for rapidly disposed compounds. By extrapolating the early plasma concentration-time data to estimate the initial concentration ($C_{0}$), NCA substantially underestimated $C_{0}$, resulting in physiologically implausible apparent initial volumes (198–320 mL/kg) that greatly exceeded the physiological rat plasma volume (40.2 mL/kg). Consequently, the NCA-derived clearance and the estimated $f_{m}$ were overestimated. In contrast, the mechanistically reconstructed clearance ($CL_{sum}$ = 81.7 mL/min/kg) yielded an $f_{m}$ value of 119 ± 20.0%, with the lower bound approaching complete conversion. Consistent with this, the PBPK model adequately reproduced the observed concentration-time profiles of both adipates when $f_{m}$ was fixed at unity (Supplementary Table S3), indicating that the apparent $f_{m}$ > 100% outcome reflects limitations of the NCA-based estimate rather than a genuine violation of mass balance. Nevertheless, the discrepancy between the reconstructed and NCA-derived clearances indicates residual uncertainty in the absolute magnitude of MEHA clearance. Therefore, model extrapolations beyond the experimental conditions evaluated in this study should be interpreted cautiously. Within the present datasets, however, the mechanistically reconstructed clearance provided a more physiologically coherent basis for prediction than the NCA-derived value.

Another important finding was that the pronounced lipophilicity of DEHA substantially influenced its absorption pathway. The GI-lymph PBPK model (Figure 2) [34] estimated that 90.0% of the absorbed DEHA entered systemic circulation through the lymphatic route. Given the estimated absorption fraction of 0.139, this corresponds to approximately 12.5% of the administered dose. Highly lipophilic compounds with a log P greater than 5 and high lipid solubility are considered favorable candidates for intestinal lymphatic transport [39,40]. For example, more than 90% of systemically available testosterone undecanoate, a lipophilic fatty-acid ester, is transported through the intestinal lymphatics in lymph-duct-cannulated dogs despite its low absolute oral bioavailability [41]. DEHA (clogP = 6.83) is a lipid-like diester with high lipophilicity, supporting the plausibility of the substantial lymphatic contribution estimated by the model. Incorporation of this lymphatic pathway was essential for reproducing the characteristic double-peak plasma PK profile and the prolonged terminal phase (i.e., flip-flop kinetics), reflecting sequential systemic entry through parallel portal-hepatic and lymphatic absorption pathways. Interestingly, a similar double-peak pattern has been reported in the urinary excretion profiles of DEHA metabolites in humans [42], although its underlying mechanism remains unclear. The present findings warrant further investigation into the potential role of lymphatic transport in DEHA absorption across species.

Beyond the absorption kinetics, the model incorporated the saturable hydrolysis of DEHA within the lymphatic compartment, enabling the simultaneous description of the mesenteric lymph concentrations of both DEHA and MEHA following oral administration (Figure 3e). Notably, the model also accurately predicted the mesenteric lymph DEHA concentration measured after IV DEHA administration (see Section 3), despite these data not being used for parameter estimation, providing independent support for the lymphatic disposition characterized by the model.

Human biomarkers of DEHA exposure include secondary oxidative metabolites, such as mono-5-carboxy-2-ethylpentyl adipate (5cx-MEPA), mono-2-ethyl-5hydroxyhexyl adipate (5OH-MEHA), and mono-2-ethyl-5-oxohexyl adipate (5oxo-MEHA), which are formed via CYP-mediated pathways [43]. However, their urinary recovery was found to be minimal (≤0.20%) in human exposure studies [42]. Consistently, only trace amounts of these metabolites (<40 ng/mL) were detected in rat plasma following the IV administration of MEHA (Supplementary Figure S2). Together with the near-complete inhibition of MEHA metabolism by BNPP (Table 1), these findings indicate that systemic MEHA clearance is predominantly governed by hydrolytic pathways rather than CYP-mediated oxidation.

This approach may also be applicable to PK analyses of soft drugs and prodrugs that undergo extensive tissue hydrolysis. Although pulmonary first-pass elimination has been recognized for a number of compounds [44,45,46], the corresponding propagation through the serial circulatory network has rarely been addressed explicitly, with most analyses relying on the simple summation of organ clearances. The present circulatory topology-based approach addresses this limitation by explicitly accounting for the shared pulmonary clearance (Supplementary Figure S1).

The current study has several limitations. First, the vascular clearance term ($CL_{T,vas})$ was inferred from the BNPP-insensitive fraction rather than measured directly. Thus, vascular lipase-mediated hydrolysis remains a mechanistic hypothesis requiring experimental confirmation. Nevertheless, the involvement of vascular hydrolysis is supported by the rapid degradation of DEHA in pancreatic and cholesterol lipase incubation systems (Table 1) and by the marked improvement in reproducing the rapid formation of MEHA after the incorporation of $CL_{T,vas}$. The remaining discrepancies in the early plasma MEHA profile (Figure 3b) suggest that additional mechanisms governing the initial phase may still require further investigation. Second, plasma MEHA concentrations following oral DEHA administration were below the analytical limit of quantification, precluding experimental verification of the model-predicted MEHA profile. Third, owing to the surgical constraints of lymph cannulation, only one mesenteric lymph sample was obtained per animal, limiting robust assessment of inter-individual variability in lymphatic transport. Fourth, the model was not validated using a fully independent external dataset, although the mesenteric lymph DEHA concentration after IV administration, which was not used for parameter estimation, was reproduced as a forward prediction. Fifth, although parameter precision and structural sensitivity were evaluated, a formal global sensitivity and uncertainty analysis was not conducted. The model code is provided to facilitate model reproduction and further sensitivity and uncertainty analyses. Finally, because esterase expression and substrate specificity differ substantially across species [47], the rat-derived kinetic parameters should be extrapolated to humans with caution.

In conclusion, the systemic PK of DEHA and MEHA was mechanistically characterized in rats using a unified PBPK approach. Extensive tissue hydrolysis, likely including vascular (lipase-mediated) metabolism, appeared to dominate the systemic clearance of both adipates, and parallel portal-hepatic and lymphatic absorption determined the prolonged oral disposition of DEHA. The present model may facilitate quantitative prediction of DEHA PK and support PBPK modeling of other rapidly hydrolyzed lipophilic ester compounds.

## Figures and Tables

**Table 1:** In vitro metabolic stability of DEHA and MEHA across various biological matrices in the presence and absence of BNPP. Experimental data are presented as mean ± standard deviation (*n* = 3).

Matrix	k (min^−1^) ^a^		% Reduction by BNPP ($f_{BNPP}\times 100$) ^c^	$t_{1/2}$ (min) ^b^	k (min^−1^) ^a^		% Reduction by BNPP ( $f_{BNPP}\times 100$) ^c^	$t_{1/2}$ (min) ^b^
	DEHA				MEHA			
	Control	+BNPP			Control	+BNPP		
Plasma	0.122 ± 0.00805	0.00160 ± 0.00269	98.7 ****	5.70	0.0316 ± 0.00417	0.0112 ± 0.00169	64.7 **	21.9
Rat liver microsome	0.0891 ± 0.00314	0.0285 ± 0.00499	68.0 ***	7.78	0.811 ± 0.0690	0.00187 ± 0.000442	99.8 **	0.854
Adipose homogenate	0.0312 ± 0.00162	0.0395 ± 0.0125	−26.7 ^ns^	22.2	2.01 ± 0.0987	0.0121 ± 0.00525	99.4 ***	0.345
Brain homogenate	0.0767 ± 0.0102	0.0775 ± 0.0212	−0.995 ^ns^	9.03	0.0572 ± 0.00907	0.00644 ± 0.00444	88.7 **	12.1
Gut homogenate	0.139 ± 0.0276	0.0957 ± 0.0170	31.2 ^ns^	4.98	8.07 ± 0.910	0.0108 ± 0.00352	99.9 **	0.0859
Heart homogenate	0.0316 ± 0.00967	0.0254 ± 0.0294	19.8 ^ns^	21.9	0.553 ± 0.0595	Stable ^d^	100 **	1.25
Kidney homogenate	0.0951 ± 0.0120	0.0283 ± 0.00303	70.2 **	7.29	4.58 ± 0.146	0.0192 ± 0.00709	99.6 **	0.151
Lung homogenate	0.0380 ± 0.00181	0.00876 ± 0.00622	76.9 *	18.2	2.01 ± 0.146	0.0192 ± 0.00143	99.0 **	0.345
Muscle homogenate	0.0322 ± 0.00280	0.0196 ± 0.0222	39.0 ^ns^	21.6	0.229 ± 0.00484	0.00327 ± 0.00398	98.6 ****	3.03
Spleen homogenate	0.0629 ± 0.00473	0.0458 ± 0.0361	27.2 ^ns^	11.0	0.275 ± 0.00675	0.000558 ± 0.000967	99.8 ***	2.52
Urine	Stable			>180	Stable			>180
FaSSGF	Stable			>180	Stable			>180
Bile	Stable			>180	Stable			>180
Mesenteric lymph	0.0122 ± 0.000300			56.8	0.146 ± 0.00242			4.73
Pancreatic lipase reaction mixture	0.224 ± 0.00532			3.09	Stable			>180
Cholesterol lipase reaction mixture	0.287 ± 0.0439			2.42	Stable			>180

^a^ Disappearance rate constants. ^b^ Half-life ($t_{1/2}=0.693/k$) calculated from the control groups. Half-lives > 180 min were considered stable. ^c^ Statistical significance between the control and BNPP-treated groups: ^ns^ *p* ≥ 0.05, * *p* < 0.05, ** *p* < 0.01, *** *p* < 0.001, **** *p* < 0.0001. ^d^ %Reduction by BNPP was essentially 100%.

**Table 2:** Summary of the $f_{u,homogenate}$, $f_{u,T}$, and $K_{pT,pred}$ values of DEHA and MEHA across nine rat tissues. Experimental data are presented as mean ± standard deviation (*n* = 3).

	DEHA			MEHA		
	$f_{u,T,homogenate}$	$f_{u,T}$	$K_{pT,pred}$	$f_{u,T,homogenate}$	$f_{u,T}$	$K_{pT,pred}$
Adipose	0.0249 ± 0.00416	0.00635	3.27	0.431 ± 0.0792	0.159	0.175
Brain	0.0170 ± 0.00527	0.00432	4.82	0.443 ± 0.188	0.166	0.167
Gut	0.164 ± 0.0225	0.0469	0.444	0.135 ± 0.0305	0.0375	0.741
Heart	0.0167 ± 0.00816	0.00422	4.93	0.355 ± 0.0564	0.121	0.230
Kidneys	0.0320 ± 0.00726	0.00819	2.54	0.684 ± 0.121	0.351	0.0792
Liver	0.0204 ± 0.00398	0.00517	4.02	0.521 ± 0.159	0.214	0.130
Lungs	0.0106 ± 0.00148	0.00268	7.76	0.131 ± 0.0131	0.0362	0.769
Muscle	0.0160 ± 0.00573	0.00405	5.13	0.446 ± 0.148	0.167	0.166
Spleen	0.0117 ± 0.00366	0.00296	7.04	0.268 ± 0.0278	0.0840	0.331

**Table 3:** Summary of the primary PK parameters calculated from NCA of the arterial plasma concentration-time data for DEHA and MEHA following their respective administration to rats. Parameter definitions are provided in the main text. Data are presented as the mean ± standard deviation.

	DEHA (IV) (*n* = 3)	MEHA (DEHA IV, *n* = 6)	MEHA (IV) (*n* = 3)	DEHA (PO) (*n* = 3)
Dose (mg/kg)	5.94	-	5	99.8
$Dose_{eff}$ (mg/kg)	5.82	-	4.79	-
AUC (ng·min/mL)	71,800 ± 17,200	60,300 ± 10,100	39,900 ± 3460	201,000 ± 100,000
$C_{max}$ (ng/mL)	7430 ± 1670	14,700 ± 2610	5080 ± 533	251 ± 67.3
CL (mL/min/kg)	84.2 ± 19.7	-	120 ± 10.6	-
CL/F (mL/min/kg)	-	-	-	586 ± 281
$V_{ss}$ (mL/kg)	959 ± 116	-	1348 ± 866	-
MRT (min)	11.8 ± 2.69	7.88 ± 2.88	11.3 ± 7.38	868 ± 291
$t_{1/2}$ (min)	20.0 ± 5.83	35.1 ± 22.7	34.7 ± 24.4	563 ± 181
$t_{max}$ (min)	2	2	2	150 ± 79.4
F *(%)*	-	-	-	17.0 ± 8.43
Estimated $f_{m}$ (%)	-	119 ± 20.0%	-	-

**Table 4:** Compound-specific parameter values for PBPK modeling of DEHA and MEHA.

Category	Parameter (Unit)	Values (CV%)					
		DEHA				MEHA	
Physicochemical properties	Molecular weight	370.6				258.4	
	Compound type	Neutral				Acid	
	pKa	-				4.11 ^a^	
	clogP	6.83 ^a^				3.66 ^a^	
	$f_{up}$	0.0208				0.0278	
	R	0.581 ^b^				0.592	
	$P_{PAMPA}$ (cm/s)	0.805 × 10^−6^ (36.1%) ^c^				48.8 × 10^−6^	
Absorption	$P_{eff}$ (cm/s) ^d^	1.35 × 10^−4^				-	
	$k_{a,DU}$ (1/min)	0.0749 ^e^				-	
	$k_{a,JE}$ (1/min)	0.153 ^e^				-	
	$k_{a,IL}$ (1/min)	0.0886 ^e^				-	
	$k_{a,CO}$ (1/min)	0.00459 ^e^				-	
	$T_{lag,LY}$ (min)	66.5 (14.2%) ^c^				-	
	$CL_{LY}$ (mL/min)	33.8 (21.4%) ^c^				-	
	$K_{m,LY}$ (ng/mL)	4640 (19.3%)				-	
	$F_{abs}$	0.139 (10.2%) ^c^				-	
Distribution /Metabolism	Tissues	$K_{pT}$	$CL_{u,T,int}$ (mL/min/kg)	$CL_{u,T,vas,int}$ (mL/min/kg)	$CL_{T,vas}$ (mL/min/kg)	$K_{pT}$	$CL_{u,T,int}$ (mL/min/kg)
	Adipose	2.77 ^h^	0	260	5.41	0.244	1240
	Bone	6.51 ^f^	-	-		0.639 ^g^	-
	Brain	3.52 ^h^	0	89.3	1.86	1.51	2.56
	Gut	0.209 ^h^	26.2	57.7	1.20	0.302	5920
	Heart	4.52 ^h^	6.31	25.5	0.531	0.275	26.2
	Kidneys	0.872 ^h^	73.1	31.0	0.645	0.0915	235
	Liver	0.742 ^h^	13,100	-	-	0.243	89,000
	Lungs	4.09 ^h^	54.5	16.4	0.341	0.860	306
	Muscle	4.74 ^h^	1460	2280	47.4	0.137	953
	Skin	22.8 ^f^	-	-	-	3.28 ^g^	-
	Spleen	2.31 ^h^	13.3	35.7	0.742	0.483	9.33
	Plasma—venous	-	157	-	-	-	30.2
	Plasma—artery	-	78.3	-	-	-	15.1
	$CL_{LU,vas}$	-	-	-	0.341 ^i^	-	-
	$\sum CL_{T,vas,art}$	-	-	-	57.8 ^i^	-	-
	$f_{m,tissue}$ (%)	-	-	-	-	-	100
Excretion	$f_{e}$ (%)	0				0	

^a^ Chemicalize (ChemAxon Ltd.); ^b^ Adjusted to the theoretical minimum value in the model (see text); ^c^ Obtained by nonlinear regression (CV% in parentheses); ^d^ Estimated using ADMET Predictor^®^ (version 12; SimulationsPlus, Inc., Lancaster, CA, USA) and extrapolated by Equations (S16) and (S17); ^e^ Equations (S18)–(S21); ^f^ Berezhkovskiy method [28]; ^g^ Schmitt method [29], using the corrected tissue-composition parameters reported in the published corrigendum; ^h^ Calculated as $K_{pT}=K_{pT,pred}\cdot K_{p,scalar}$, where $K_{p,scalar}$ = 0.542 (60.0%; see text); ^i^ $CL_{LU,vas}$ denotes $CL_{T,vas}$ in the lungs, whereas $\sum CL_{T,vas,art}$ denotes the sum of $CL_{T,vas}$ values in all other tissues.

**Table 5:** Circulatory topology-based clearance values for DEHA and MEHA with respect to arterial plasma. $CL_{path}$ values were estimated from in vitro tissue metabolic stability data using Equations (12)–(17).

$CL_{path}$ **for N on-Splanchnic Pathways (mL/min/kg)**	**DEHA**	**MEHA**
Adipose	0.233	8.57
Brain	0.0555	0.179
Heart	0.267	0.959
Kidneys	1.57	6.22
Muscle	12.7	17.9
Skin	0.253	0.514
Bone	0.500	1.02
Rest of body	0.289	0.589
$CL_{path}$ **for splanchnic pathways (mL/min/kg)**	**DEHA**	**MEHA**
Liver	3.00	24.2
Gut—Liver	2.75	19.1
Spleen—Liver	0.319	2.05
**Components of** $CL_{sum}$ **(mL/min/kg)**	**DEHA**	**MEHA**
$\sum CL_{path}$ (all pathways)	22.0	81.3
$\sum CL_{T,vas,art}$ (Table 4)	57.8	0
$CL_{p,art}$ (Equation (9))	1.63	0.420
$CL_{sum}$ (Equation (11))	81.3	81.7
CL (NCA; Table 3)	84.2 ± 19.7	120 ± 10.6

## Data Availability

The original data presented in the study are openly available in Zenodo at https://doi.org/10.5281/zenodo.21019549.

## Supplementary Materials

The following supporting information can be downloaded at: https://www.mdpi.com/article/10.3390/pharmaceutics18081001/s1. Table S1: Systemic physiological variables of a 250 g rat; Table S2: Tissue variables of a 250 g rat; Table S3: Summary of the predictive performance of the PBPK model; Table S4: Parameter correlation matrices from the maximum-likelihood estimation (ADAPT 5); Figure S1: Sensitivity of systemic clearance estimation to pulmonary availability ($F_{LU}$); Figure S2: Plasma concentration–time profiles of secondary metabolites of DEHA following intravenous administration of MEHA (5 mg/kg) in rats. Supplementary Material File S1: “DEHA_PBPK_model_BM code”.
