# Supplementary material for: Physiologically Based Pharmacokinetic Modeling of Di(2-ethylhexyl) Adipate and Its Primary Metabolite, Mono(2-ethylhexyl) Adipate, in Rats, Incorporating Circulatory Topology-Based Multi-Tissue Metabolism and Lymphatic Absorption
Source: Pharmaceutics. 2026 Aug 13;18(8):1001. doi: 10.3390/pharmaceutics18081001 (PMC13516969; doi:10.3390/pharmaceutics18081001)

Supplementary Material

Physiologically Based Pharmacokinetic Modeling of Di(2-ethylhexyl) Adipate and Its Primary Metabolite Mono(2-ethylhexyl) Adipate in Rats, Incorporating Circulatory Topology-Based Multi-Tissue Metabolism and Lymphatic Absorption

Eunsuk Yang, Yoo-Seong Jeong, Minsang Kim, Seungchan Kim, and Suk-Jae Chung*

College of Pharmacy, Seoul National University, Seoul 08826, Republic of Korea

Materials and Reagents

Deuterium-labeled DEHA (DEHA-d8), in which all eight hydrogen atoms on the adipate chain were replaced with deuterium, was purchased from Toronto Research Chemicals (Toronto, Canada). Unlabeled DEHA, phenylmethylsulfonyl fluoride (PMSF), bis-(p-nitrophenyl) phosphate (BNPP), β-glucuronidase with residual sulfatase activity from *Helix pomatia*, lipase from porcine pancreas, cholesterol lipase from porcine pancreas, sodium taurocholate, dimethyl sulfoxide (DMSO; molecular biology grade), potassium chloride, valsartan, Cremophor EL, formic acid, and ethanol (molecular biology grade) were obtained from Sigma-Aldrich (St Louis, MO, USA). Rapid equilibrium dialysis (RED) base plates and inserts and HPLC-grade acetonitrile and methanol were purchased from Thermo Fisher Scientific (Waltham, MA, USA). Dulbecco's phosphate-buffered saline (DPBS) was purchased from Welgene (Gyeongsan, Korea), and polyethylene glycol 400 (PEG400) was purchased from Duksan Pure Chemicals (Ansan, Korea). Normal saline was purchased from JW Pharmaceutical (Seoul, Korea). Zoletil® 50 (Virbac, Carros, France) and Rompun® (Bayer, North Rhine-Westphalia, Germany) were used in this study. Rat liver microsomes and reagents for the nicotinamide adenine dinucleotide 2'-phosphate (NADPH) regenerating system were purchased from Corning (Woburn, MA, USA). Fasted-state simulated gastric fluid (FaSSGF) was purchased from Biorelevant (London, UK). MEHA was custom-synthesized by J Mackem (Seoul, Korea). All reagents were used without further purification.

Quantitative analysis of DEHA and its metabolites

The concentrations of DEHA-d8, DEHA, and MEHA in *in vivo* and *in vitro* samples were quantified using an HPLC system (Agilent 1200 series, Agilent, Santa Clara, CA, USA) coupled with an MS/MS system (API 4000 mass spectrometer, Applied Biosystems, Foster City, CA, USA). Sample aliquots (10 μL for mesenteric lymph and 50 μL for all other samples) were mixed with ice-cold acetonitrile (for DEHA-d8 and DEHA) or methanol (for MEHA) containing valsartan (100 ng/mL) as an internal standard (90 μL for mesenteric lymph and 200 μL for other samples). After vortex-mixing for 10 min and centrifugation at 16,100 g and 4°C for 5 min, the supernatant was collected, and a 10-μL aliquot was directly injected into the HPLC-MS/MS system.

Chromatographic separation was performed on a Phenomenex Luna CN column (3 μm, 100 Å, 50 × 2 mm). Mobile phase A was 0.1% (v/v) formic acid in acetonitrile for DEHA-d8 and DEHA analyses, and 0.1% (v/v) formic acid in methanol for MEHA analysis. Mobile phase B was 0.1% (v/v) formic acid in purified water.

The analytes and internal standard were eluted at a flow rate of 0.4 mL/min using a gradient program starting at 10% mobile phase A; increasing to 90% over 2 min; decreasing to 10% over 1 min; and held for 5 min.

Samples were ionized using a turbo ion spray interface and analyzed in the multiple reaction monitoring (MRM) mode. DEHA, DEHA-d8, and valsartan were monitored in the positive ionization mode, whereas MEHA and valsartan were monitored in the negative ionization mode. The monitored Q1/Q3 transitions (m/z) were 371.5/128.9 for DEHA, 379.4/137.0 for DEHA-d8, 256.9/82.8 for MEHA, 436.2/234.9 for valsartan in the positive ion mode, and 434.1/178.8 for valsartan in the negative ion mode. The common source parameters as follows (values in parentheses): curtain gas pressure (20 psi), source temperature (500°C), ion source gas 1 (50 psi), ion source gas 2 (55 psi), and ion spray voltage (5000 V in the positive mode and −4500 V in the negative mode). The declustering potentials were set at 91 V for DEHA, 106 V for DEHA-d8, -80 V for MEHA, 91 V (positive mode) or -80 V (negative mode) for valsartan. The entrance potentials were set at 10 V for DEHA, DEHA-d8, and valsartan in the positive mode, and −10 V for MEHA and valsartan in the negative mode. The collision energies were set at 21 V for DEHA, 23 V for DEHA-d8, -26 V for MEHA, and 25 V (positive mode) or -30 V (negative mode) for valsartan. The collision cell exit potentials were set at 8 V for DEHA, 14 V for DEHA-d8, -7 V for MEHA, 16 V (positive mode) or -11 V (negative mode) for valsartan.

Details of the *in vivo* PK Studies in Rats: Intravenous Administration

Male Sprague-Dawley rats were anesthetized by intramuscular administration of Zoletil® 50 (16 mg/kg tiletamine and 16 mg/kg zolazepam) and Rompun® (3.7 mg/kg xylazine HCl). Under anesthesia, surgical procedures appropriate for each experimental protocol were performed. For IV administration and/or arterial blood sampling, the femoral vein and artery were catheterized with polyethylene tubing (SP-45; inner diameter, 0.58 mm; outer diameter, 0.86 mm; Natsume Corporation, Tokyo, Japan). In all studies, the total blood volume withdrawn was kept below 2 mL per animal (i.e., < 10% of the total blood volume of a 250-g rat).

For IV bolus administration, rats received either DEHA (3 mg/kg unlabeled plus 3 mg/kg DEHA-d8, corresponding to 5.94 mg/kg as DEHA equivalents) or MEHA (5 mg/kg). Both adipates were prepared in a vehicle consisting of DMSO, Cremophor EL, PEG400, and normal saline (10:5:35:50, v/v; 2 mL/kg), and injected through the venous catheter over 5 s. Blood samples (200 µL) were collected from the femoral artery into heparinized syringes at 2, 5, 15, 30, 60, 90, 120, 240, and 480 min post-dose. Plasma was obtained by centrifugation of blood

samples at 16,100 g for 5 min at 4°C. Following IV administration of DEHA, plasma concentrations of DEHA-d8 and MEHA formed from unlabeled DEHA were quantified. The measured DEHA-d8 concentrations (molecular weight: 378.6) were converted to DEHA-equivalent concentrations (molecular weight: 370.6). The measured unlabeled MEHA concentrations were corrected to represent total MEHA-equivalent concentrations based on the molecular weight difference between unlabeled MEHA (258.4) and MEHA-d8 (266.5).

For the determination of urinary and biliary excretion, separate groups of rats received the same IV doses of DEHA (5.94 mg/kg) or MEHA (5 mg/kg). Urine was collected in metabolic cages over a 24-h period. Bile was collected through a bile duct cannula at 10-min intervals for up to 120 min after dosing. These sampling durations were considered sufficient based on the short terminal half-lives of both compounds. To quantify glucuronide and sulfate conjugates, each urine and bile sample was divided into two aliquots: one aliquot was analyzed directly, whereas the other was subjected to enzymatic hydrolysis with β-glucuronidase/sulfatase prior to analysis.

Details of the *in vivo* PK Studies in Rats: Oral or Intralymphatic Administration

For oral administration, unlabeled DEHA (90 mg/kg) and DEHA-d8 (10 mg/kg), corresponding to 99.8 mg/kg as DEHA equivalents, were dissolved in soybean oil (2 mL/kg). Blood samples (100 μL) were collected from the jugular vein into heparinized syringes at 15, 30, 60, 90, 120, 240, 360, 480, and 720 min post-dose. Plasma was obtained by centrifugation of blood samples at 16,100 g for 5 min at 4°C. Mesenteric lymph was collected according to the method of Porter and Charman [17], with minor modifications: Following lymphatic cannulation, mesenteric lymph was continuously collected through the catheter at a flow rate of approximately 0.6 mL/h, and lymph samples were obtained at a single time point (30, 60 or 90 min post-dose). Plasma and mesenteric lymph samples were analyzed for both DEHA and MEHA. Different vehicles were used for the IV and oral studies because of the poor aqueous solubility of DEHA and the approximately 17-fold higher oral dose. The IV cosolvent/surfactant vehicle could not accommodate the oral dose within the dosing volume of 2 mL/kg. Therefore, soybean oil was used for oral administration. Because a lipid vehicle may influence intestinal solubilization and lymphatic uptake, the oral PK results represent absorption under this lipid-based dosing condition. This condition is relevant to dietary exposure because DEHA preferentially migrates from food-contact materials into lipid-rich foods, so that human oral exposure occurs predominantly through the ingestion of DEHA

already dissolved in dietary lipids. The lipid-based oral administration used here (soybean oil) therefore closely reproduces the physical form in which DEHA is realistically ingested.

Determination of the Unbound Fraction in Plasma (f_{up}) and Blood-to-Plasma Partition Coefficient (R) of DEHA and MEHA

The unbound fraction of DEHA in rat plasma (f_{up}) was determined using solid-phase microextraction (SPME) according to Musteata*, et al.* [22] with minor modifications. Plasma samples containing DEHA were prepared at a final concentration of 10 μM, and DPBS calibration solutions containing DEHA ranging from 0.1 to 10 μM were prepared in parallel. BNPP (1 mM) was added to the plasma to prevent hydrolysis [14]. After a 1-h incubation, SPME fibers were desorbed with 200 μL of 80% methanol for 30 min. The unbound plasma concentrations of DEHA were determined using a calibration curve constructed from the relationship between DEHA concentrations in DPBS and the corresponding SPME desorption concentrations.

The f_{up} of MEHA was determined using rapid equilibrium dialysis (RED) according to the manufacturer's protocol [21]. Before use, the RED device was rinsed with 20% ethanol for 10 min. MEHA was spiked into rat plasma at a final concentration of 2000 ng/mL, and BNPP (1 mM) was added to prevent hydrolysis during the assay. A 200-μL aliquot of the spiked plasma and 350 μL of DPBS were added to the sample and buffer chambers. The device was sealed and incubated on an orbital shaker (100 rpm) at 37°C for 4 h. After incubation, aliquots were collected from both chambers and analyzed for MEHA by LC-MS/MS. The unbound fraction was calculated as the ratio of the MEHA concentration in the buffer chamber to that in the plasma chamber.

For determination of the blood-to-plasma partition coefficient (R), freshly heparinized blood (25 IU/ml) was collected from anesthetized male SD rats via cardiac puncture. Stock solutions of DEHA or MEHA in DMSO were added to achieve a final concentration of 10 μM. BNPP (1 mM) was added to blood to prevent hydrolysis. The final DMSO concentration was maintained below 0.1% to avoid hemolysis. After a 30-min incubation, DEHA or MEHA concentrations in whole blood and plasma were measured to calculate the R values.

PBPK Model Equations

The absorption and disposition kinetics of DEHA and MEHA were described by a whole-body PBPK

model (Figure 2), consisting of eleven tissue compartments (abbreviated in parentheses): adipose (AD), bone (BO), brain (BR), gut (GU), heart (HE), kidney (KI), liver (LI), lung (LU), muscle (MU), skin (SK), and spleen (SP) plus arterial blood ($art, blood$) and venous blood ($ven, blood$) pools, with an additional lymph (LY) compartment. The PBPK model equations are presented in a generalized form, where the subscript T denotes a generic tissue compartment unless otherwise specified. This notation applies to both DEHA and MEHA.

The disposition kinetics of DEHA and MEHA within a tissue were described by the capillary permeability model [38], which was further extended to incorporate peripheral tissue elimination [33]. For each tissue, the permeability-surface area product (PS_T) was calculated as:

$$PS_T = P_{PAMPA} \cdot S_{eff,T} \tag{S.1}$$

where P_{PAMPA} is the PAMPA permeability coefficient and $S_{eff,T}$ is the effective surface area of the tissue (Supplementary Table S2), taken from the literature [38]. An experimental value was obtainable for MEHA, whereas a model-fitted value was estimated for DEHA (see the main text).

Based on the calculated PS_T, the tissue permeability limitation was described using the fractional distribution parameter of the tissue ($f_{d,T}$; identical to f_d in the main text), expressed as:

$$f_{d,T} = 1 - \exp\left(-\frac{f_{up} \cdot PS_T}{Q_T \cdot R}\right) \tag{S.2}$$

where f_{up} is the unbound fraction in plasma, R is the blood-to-plasma partition coefficient, and Q_T is the blood flow to the tissue. Conceptually, $f_{d,T}$ represents the fraction of drug that effectively permeates into the tissue cells during a single pass through the capillary. When PS_T is sufficiently large relative to tissue blood flow, $f_{d,T}$ approaches unity corresponding to perfusion-limited distribution. Conversely, when PS_T is small, $f_{d,T}$ approaches $f_{up}PS_T/(Q_T R)$ (i.e., permeability-limited).

The drug concentration in blood leaving the tissue ($C_{out,T}$) is defined as:

$$C_{out,T} = \left(1 - f_{d,T}\right) \cdot C_{in,T} + f_{d,T} \cdot \frac{C_T \cdot R}{K_{p,T}} \tag{S.3}$$

where $C_{in,T}$ is the inflow blood concentration, $K_{p,T}$ is the tissue-to-plasma partition coefficient, and C_T is the tissue concentration. Eq. (S.3) indicates that $C_{out,T}$ consists of two components: a fraction that bypasses tissue partitioning [$(1 - f_{d,T}) \cdot C_{in,T}$] and a fraction that equilibrates with the tissue ($f_{d,T} \cdot C_T R/K_{p,T}$). The product of

$C_{out,T}$ and the corresponding Q_T determines the rate of drug leaving the tissue and entering the downstream blood pools.

Melding Eq. (S.3) into Fick's law of perfusion $[dA/dt = Q_T \cdot (C_{in,T} - C_{out,T})]$ yields the differential equation describing the rate of change in drug amount within the tissue. For most tissues receiving arterial blood flow (e.g., adipose, bone, brain, heart, kidney, muscle, skin, and spleen), $C_{in,T}$ is defined as the arterial blood concentration, $C_{art,blood}$. Therefore, the corresponding differential equation is written as:

$$V_T \frac{dC_T}{dt} = Q_T \cdot f_{d,T} \cdot \left(C_{art,blood} - \frac{C_T \cdot R}{K_{p,T}}\right) - CL_{u,T,int} \cdot f_{up} \cdot \frac{C_T}{K_{p,T}} + R_{form,T} \quad \text{(S.4)}$$

where V_T is the anatomical tissue volume, $CL_{u,T,int}$ is the intrinsic clearance occurring within the tissue parenchyma, acting upon the unbound tissue concentration, and $R_{form,T}$ is the source term representing MEHA formation from DEHA, which is set to zero for DEHA. The mathematical description of $R_{form,T}$ for each tissue is summarized below.

The differential equation for the gut compartment was obtained by extending the generic tissue equation with two additional processes:

$$V_{GU} \frac{dC_{GU}}{dt} = Q_{GU} f_{d,GU} \left(C_{art,blood} - \frac{C_{GU} \cdot R}{K_{p,GU}}\right) - CL_{u,GU,int} f_{up} \frac{C_{GU}}{K_{p,GU}} + R_{form,GU} + F_{abs} \cdot f_{dis} \sum k_{a,j} X_j - CL_{LY} \cdot C_{GU} \quad \text{(S.5)}$$

where $F_{abs} \cdot f_{dis} \sum k_{a,j} X_j$ represent the dissolution and absorption input of DEHA from the intestinal segments, aggregated from the duodenum, jejunum, ileum, and colon compartments (see below). The term $CL_{LY} \cdot C_{GU}$ represents the lymphatic uptake of DEHA from the gut, which enters the lymphatic compartment described as:

$$V_{LY} \frac{dC_{LY}}{dt} = CL_{LY} \cdot C_{GU} - Q_{LY} \cdot C_{LY} - k_{LY} \cdot V_{LY} \cdot C_{LY} + R_{form,lymph} \quad \text{(S.6)}$$

where $Q_{LY} \cdot C_{LY}$ represents the transport rate of DEHA returning to the venous blood via the lymph flow Q_{LY}, and the term $k_{LY} \cdot V_{LY} \cdot C_{LY}$ describes its hydrolysis within the lymphatic compartment. The lymphatic hydrolysis rate constant k_{LY} was, however, treated differently for the two compounds. Because the lymphatic DEHA concentrations observed *in vivo* were high (reaching approximately 100,000 ng/mL), at which the hydrolysis may become saturable, k_{LY} for DEHA was assumed to follow Michaelis–Menten kinetics, expressed as $k_{LY} = \frac{V_{max,LY}}{K_{m,LY} + C_{LY}}$. In contrast, we hypothesized that compared to DEHA, the lymphatic MEHA concentrations may fall

below the range where saturation would be negligible: Based on this reasoning, k_{LY} for MEHA was described as the first-order rate constant and fixed at the *in vitro* observed value.

The liver receives blood from three inflow streams: the hepatic artery ($Q_{LI} - Q_{GU} - Q_{SP}$), together with the venous outflows from the gut and spleen (Q_{GU} and Q_{SP}). Accordingly, the inflow concentration ($C_{in,LI}$) is defined as:

$$C_{in,LI} = \frac{(Q_{LI}-Q_{GU}-Q_{SP})\cdot C_{art,blood}+Q_{GU}\cdot C_{out,GU}+Q_{SP}\cdot C_{out,SP}}{Q_{LI}} \tag{S.7}$$

which is used to describe the differential equation for the liver as:

$$V_{LI}\frac{dC_{LI}}{dt} = Q_{LI}f_{d,LI}\left(C_{in,LI} - \frac{C_{LI}\cdot R}{K_{p,LI}}\right) - CL_{u,LI,int}\cdot f_{up}\cdot\frac{C_{LI}}{K_{p,LI}} + R_{form,LI} \tag{S.8}$$

where $CL_{u,LI,int}$ is the hepatic intrinsic clearance (see the main text).

Given that the lung is perfused by the entire cardiac output (Q_{CO}) draining from the venous blood pool, $C_{in,LU}$ is defined as $C_{ven,blood}$, and Q_{LU} is equal to Q_{CO}. The differential equation for the lung therefore follows the same mass balance form as the generic tissue [Eq. (S.4)], expressed as:

$$V_{LU}\frac{dC_{LU}}{dt} = Q_{CO}f_{d,LU}\left(C_{ven,blood} - \frac{C_{LU}\cdot R}{K_{p,LU}}\right) - CL_{u,LU,int}f_{up}\frac{C_{LU}}{K_{p,LU}} + R_{form,LU} \tag{S.9}$$

where $CL_{u,LU,int}$ is the pulmonary intrinsic clearance.

The venous blood pool collects the outflows from the non-splanchnic tissues (summed over $T = AD$, BO, BR, HE, KI, MU, and SK), the liver ($Q_{LI}\cdot C_{out,LI}$), the residual cardiac output returning directly without passing through a tissue compartment ($Q_{rest}\cdot C_{art,blood}$), and the delayed lymphatic return, $Q_{LY}\cdot C_{LY}(t - T_{lag,LY})$. Drug leaves the venous blood pool through the entire cardiac output to the lung ($Q_{CO}\cdot C_{ven,blood}$) and is subject to the venous plasma clearance ($CL_{p,ven}$) and the lung vascular clearance ($CL_{LU,vas}$). $Input_{IV}$ denotes the IV dose administered into the venous blood. The differential equation for the venous blood pool is expressed as:

$$V_{ven}\frac{dC_{ven,blood}}{dt} = \Sigma Q_T C_{out,T} + Q_{LI}\cdot C_{out,LI} + Q_{rest}\cdot C_{art,blood} + Q_{LY}\cdot C_{LY}\left(t - T_{lag,LY}\right) - Q_{CO}\cdot C_{ven,blood} - \left(CL_{p,ven} + CL_{LU,vas}\right)\cdot\frac{C_{ven,blood}}{R} + R_{form,ven} + Input_{IV} \tag{S.10}$$

The arterial blood pool receives the lung outflow ($Q_{CO}\cdot C_{out,LU}$) and distributes the cardiac output to

the tissues ($Q_{CO} \cdot C_{art,blood}$). The arterial plasma clearance ($CL_{p,art}$) and the sum of tissue vascular clearances for all tissues other than the lung ($\sum CL_{T,vas,art}$) act upon the arterial plasma concentration ($C_{art,blood}/R$). The differential equation for the arterial blood pool is expressed as:

$$V_{art}\frac{dC_{art,blood}}{dt} = Q_{CO} \cdot \left(C_{out,LU} - C_{art,blood}\right) - \left(CL_{p,art} + CL_{T,vas,art}\right) \cdot \frac{C_{art,blood}}{R} + R_{form,art} \quad \text{(S.11)}$$

PBPK Parameters Related to the Metabolic Conversion of DEHA to MEHA

The rate of MEHA formation from DEHA in each compartment is represented by the source term R_{form}. For DEHA, R_{form} is zero. For MEHA, R_{form} is equal to the corresponding DEHA elimination rate scaled by the molecular-weight ratio (MW_{MEHA} / MW_{DEHA}), assuming complete conversion (f_m = 1). R_{form} is defined for the tissues (Eq. (S.12)), the arterial blood (Eq. (S.13)), and the venous blood (Eq. (S.14)), expressed as:

$$R_{form,T} = CL_{u,T,int} \cdot f_{up,DEHA} \cdot \frac{C_{T,DEHA}}{K_{p,T,DEHA}} \cdot \frac{MW_{MEHA}}{MW_{DEHA}} \quad \text{(S.12)}$$

$$R_{form,art} = \left(CL_{p,art} + CL_{T,vas,art}\right) \cdot \frac{C_{art,blood,DEHA}}{R} \cdot \frac{MW_{MEHA}}{MW_{DEHA}} \quad \text{(S.13)}$$

$$R_{form,ven} = \left(CL_{p,ven} + CL_{LU,vas}\right) \cdot \frac{C_{ven,blood,DEHA}}{R} \cdot \frac{MW_{MEHA}}{MW_{DEHA}} \quad \text{(S.14)}$$

Eqs. (S.13) and (S.14) describe MEHA formation within the arterial and venous blood through plasma- and vascular (lipase-mediated) hydrolysis of DEHA, which constitutes the major source of rapid early-phase MEHA formation in the present model. All clearance terms in Eqs. (S.12) to (S.14), i.e., $CL_{u,T,int}$ (the tissue parenchymal intrinsic clearance), $CL_{u,T,vas,int}$ (the microvascular intrinsic clearance), $CL_{T,vas}$ (the microvascular clearance), $CL_{p,art}$ and $CL_{p,ven}$ (the arterial and venous plasma clearances), are defined in the main text.

Absorption-Related PBPK Parameters

The oral absorption of DEHA through the portal and lymphatic pathways was described using the gastrointestinal (GI)-lymph model adapted from Tao et al. (2025). The corresponding equations are provided below. For Weibull dissolution, the dissolution half-life (α = 1 min), shape parameter (β = 1), and lag time ($T_{lag,dis}$ = 0.1 min) were considered to represent immediate drug release and dissolution:

$$f_{dis} = 1 - \exp\left(-\frac{(t - T_{lag,dis})^{\beta}}{\alpha}\right) \tag{S.15}$$

which was applied in Eq. (S.5).

Based on the physicochemical property of DEHA (Table 4), a Caco-2 permeability value (P_{Caco-2}) was predicted using ADMET® Predictor 12 (SimulationsPlus, Inc.), and used to estimate the human effective intestinal permeability of DEHA ($P_{eff,man}$) based on:

$$P_{eff,man} = 0.4926 \cdot \log P_{Caco-2} - 0.1454 \tag{S.16}$$

where $P_{eff,man}$ is in a unit of 10^{-4} cm/s, and P_{Caco-2} is in a unit of 10^{-6} cm/s. The resulting $P_{eff,man}$ was used to estimate the rat effective intestinal permeability of DEHA ($P_{eff,rat}$, in a unit of 10^{-6} cm/s) using:

$$P_{eff,rat} = \frac{P_{eff,man} - 0.03}{3.6} \tag{S.17}$$

which is denoted as P_{eff} hereafter.

Assuming a cylindrical geometry, the first-order absorption rate constants (k_a) specific for duodenum (DU), jejunum (JE), ileum (IL), and colon (CO), were calculated from P_{eff} and the intestinal radius (r = 0.18 cm) [15], with the degree of flatness coefficient (DF) of 1.7 and the ratio of surface area amplification factors of each segment to that of the jejunum ($SAEF_j$ = 0.49 for DU; 1 for JE; 0.58 for IL; and 0.03 for CO) [34]:

$$k_{a,DU} = \frac{2 \cdot DF \cdot P_{eff} \cdot SAEF_{DU}}{r} \tag{S.18}$$

$$k_{a,JE} = \frac{2 \cdot DF \cdot P_{eff} \cdot SAEF_{JE}}{r} \tag{S.19}$$

$$k_{a,IL} = \frac{2 \cdot DF \cdot P_{eff} \cdot SAEF_{IL}}{r} \tag{S.20}$$

$$k_{a,CO} = \frac{2 \cdot DF \cdot P_{eff} \cdot SAEF_{CO}}{r} \tag{S.21}$$

The transit, absorption, and elimination of DEHA along the GI tract were described by:

$$\frac{dX_{ST}}{dt} = -k_{ST} \cdot X_{ST} \tag{S.22}$$

$$\frac{dX_{DU}}{dt} = k_{ST} \cdot X_{ST} - f_{dis} \cdot k_{a,DU} \cdot X_{DU} - k_{DU} \cdot X_{DU} \tag{S.23}$$

$$\frac{dX_{JE}}{dt} = k_{DU} \cdot X_{DU} - f_{dis} \cdot k_{a,JE} \cdot X_{JE} - k_{JE} \cdot X_{JE} \tag{S.24}$$

$$\frac{dX_{IL}}{dt} = k_{JE} \cdot X_{JE} - f_{dis} \cdot k_{a,IL} \cdot X_{IL} - k_{IL} \cdot X_{IL} \tag{S.25}$$

$$\frac{dX_{CO}}{dt} = k_{IL} \cdot X_{IL} - f_{dis} \cdot k_{a,CO} \cdot X_{CO} - k_{CO} \cdot X_{CO} \tag{S.26}$$

$$\frac{dX_{Fe}}{dt} = k_{CO} \cdot X_{CO} \tag{S.27}$$

where X_j is the amount of DEHA in segment j (i.e., stomach, ST; duodenum, DU; jejunum, JE; ileum, IL; colon, CO, and feces, FE), and k_j is the transit rate constant of each segment (Supplementary Table S1).

Supplementary Table S1. Systemic physiological variables of a 250-g rat [Simcyp® Simulator V23 rat module, Certara UK Ltd.; Turner and Barrowman [35]; Tao et al. [34]

Parameter (unit)	Value
Cardiac output (mL/min)	80
Lymph flow (mL/min)	0.0417
Hematocrit	0.418
k_{ST} (1/min)	0.0240
k_{DU} (1/min)	0.0410
k_{JE} (1/min)	0.0137
k_{IL} (1/min)	0.0137
k_{CO} (1/min)	0.00267

Supplementary Table S2. Tissue variables of a 250-g rat [Simcyp® Simulator V23 rat module, Certara UK Ltd.; Jeong et al.[38]]

	**Blood Flow (mL/min)**	**Tissue Volume (mL)**	S_{eff} **(m²/g tissue)**
Adipose	4.72	16.66	3.02
Bone	8.08	15.68	2.34
Brain	1.12	1.24	1.67
Gut	8.08	6.19	16.1
Heart	3.2	1.05	18.1
Kidneys	11.6	2.19	37.4
Liver	19.36	8.57	14.8
Lungs	80	1.24	14.4
Muscle	18.96	116.13	5.71
Skin	4.08	39.41	0.843
Spleen	0.88	0.57	4.56
Vein	80	11.49	-
Artery	80	5.75	-
Plasma	-	10.04	-
Lymph	-	17.24	-
RBC	-	7.2	-

Supplementary Table S3. Summary of the predictive performance of the PBPK model based on MSLD, AAFE, and exposure metrics (C_{max} and AUC).

Administered adipate (its route of administration)	PK datasets	MSLD	AAFE	C_{max}		AUC	
				Obs	Pred	Obs	Pred
DEHA (IV)	DEHA plasma concentration	0.230	2.67	7430 ± 1670	2210 (2m) 98,150 (max)	71,800 ± 17,,200	72,,100
	MEHA plasma concentration	0.188	2.46	14,700 ± 2610	3750 (2m) 19,700 (max)	60,300 ± 10,100	43800
DEHA (oral)	DEHA plasma concentration	0.0157	1.27	227 ± 60.7	214	196,000 ± 91,900	128,00
	DEHA lymph concentration	0.0661	1.79	75,500 ± 24,200	106,000	-	-
	MEHA lymph concentration	0.0117	1.23	336 ± 165		-	-
MEHA (IV)	MEHA plasma concentration	0.00469	1.15	5083 ± 533	4320(2m) 81,400(max)	39,900 ± 3460	58,100

Supplementary Table S4. Parameter correlation matrices from the maximum-likelihood estimation (ADAPT 5). (A) Disposition parameters ($P_{PAMPA,DEHA}$and $K_{p,scalar}$) estimated by simultaneously fitting the plasma DEHA data following IV DEHA administration and the plasma MEHA data formed after IV DEHA administration. (B) Oral absorption and lymphatic parameters (CL_{LY}, $T_{lag,LY}$, $K_{m,LY}$, and absorbed fraction (F_{abs})) estimated from the plasma and mesenteric lymph concentration-time data following oral DEHA administration, with the disposition parameters fixed.

(A)

	$P_{PAMPA,DEHA}$	$K_{p,scalar}$
$P_{PAMPA,DEHA}$	1.00	
$K_{p,scalar}$	0.75	1.00

(B)

	CL_{LY}	$T_{lag,LY}$	$K_{m,LY}$	F_{abs}
CL_{LY}	1.00			
$T_{lag,LY}$	-0.16	1.00		
$K_{m,LY}$	0.11	0.07	1.00	
F_{abs}	0.29	0.16	0.28	1.00

Supplementary Figure S1. Sensitivity of systemic clearance estimation to pulmonary availability (F_{LU}). The clearance ratio R_{CL} was defined as the ratio of the systemic clearance obtained by conventional parallel summation of individual tissue clearances (CL_T from Eq. (7)) to that calculated using the circulatory topology-based model (CL_{sum}). F_{LU} was varied because the lung is common to all circulatory pathways, and therefore governs the extent of serial elimination. The solid and dashed lines represent DEHA and MEHA, respectively. As F_{LU} decreased, R_{CL} for DEHA remained close to unity because its systemic clearance was dominated by arterial vascular metabolism, whereas R_{CL} for MEHA increased progressively above unity, indicating that conventional parallel summation increasingly overestimates systemic clearance when pulmonary extraction becomes substantial.

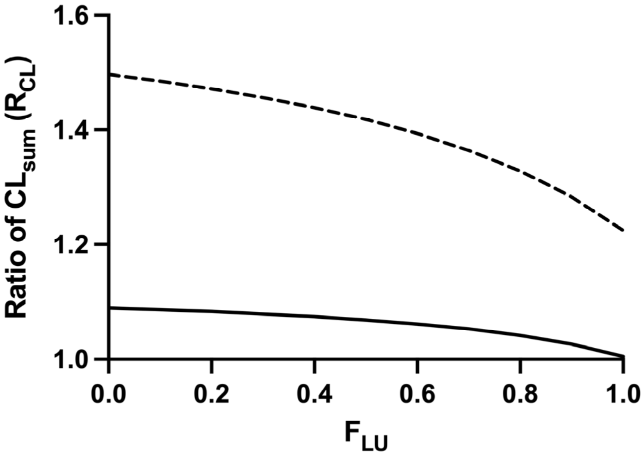

Supplementary Figure S2. Plasma concentration–time profiles of secondary metabolites of DEHA following intravenous administration of MEHA (5 mg/kg) in rats.

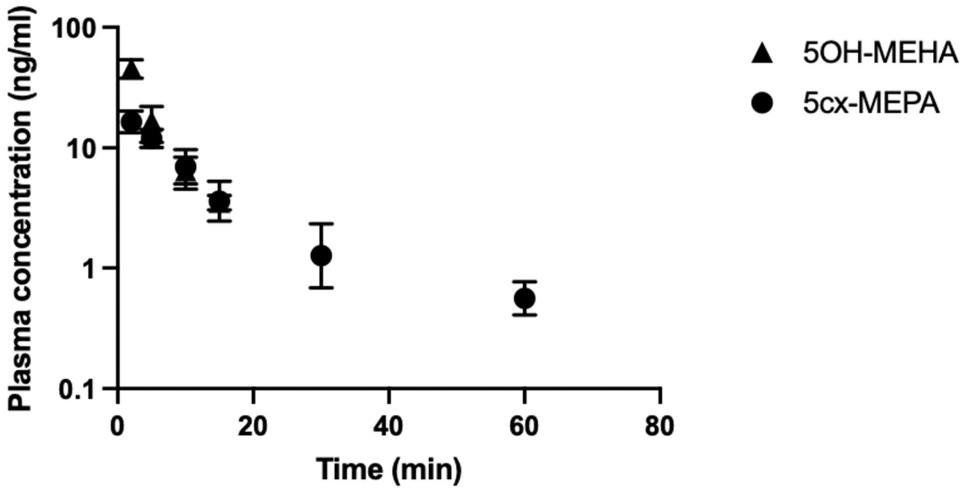

Supplement: Supplementary file 1 [file pharmaceutics-18-01001-s001.zip › pharmaceutics-4435614-supplementary material file 1.pdf]
