# Supplementary material for: Physiologically Based Pharmacokinetic Modeling of Di(2-ethylhexyl) Adipate and Its Primary Metabolite, Mono(2-ethylhexyl) Adipate, in Rats, Incorporating Circulatory Topology-Based Multi-Tissue Metabolism and Lymphatic Absorption
Source: Pharmaceutics. 2026 Aug 13;18(8):1001. doi: 10.3390/pharmaceutics18081001 (PMC13516969; doi:10.3390/pharmaceutics18081001)
Supplement: Supplementary file 1 [file pharmaceutics-18-01001-s001.zip › pharmaceutics-4435614- DEHA_PBPK_model_BM code.pdf]

Physiologically Based Pharmacokinetic Modeling of Di(2-ethylhexyl) Adipate and Its Primary Metabolite Mono(2-ethylhexyl) Adipate in Rats, Incorporating Circulatory Topology-Based Multi-Tissue Metabolism and Lymphatic Absorption

Berkeley Madonna model code

```
METHOD RK4

STARTTIME = 0
STOPTIME = 1000
DT = 0.001
DTOUT = 0.1

{dose}
;;IV;;
dose_IV_DEHA = 0;5.94 * 1000 * 1000 * BW/1000 ;ng
dose_IV_MEHA = 0;5 * 1000 * 1000 * BW/1000    ;ng

;;PO;;
dose_PO_DEHA = 99.8 * 1000 * 1000 * BW/1000 ;ng


{fitted value}
T_lag_lymph = 66.48311772 ; fitted
CL_lymph = 33.79193675 ; fitted


P_PAMPA_DEHA = 8.050915946e-07 ; fitted
Kp_scaler_DEHA = 0.5419000121 ; fitted
```

```
PO_scaler_DEHA = 0.1391578457       ; fitted
Km_lymph_DEHA = 4643.08215          ; fitted



;;;;;;;;;;;;;;;;;;;;;;;;;
;;;;;;;;;;DEHA;;;;;;;;;;
;;;;;;;;;;;;;;;;;;;;;;;;;

{administration}
dosing_time_IV = 5/60 ;min
IV_input_DEHA = IF t< dosing_time_IV THEN dose_IV_DEHA/dosing_time_IV ELSE 0

{oral absorption model}

Frac_dis_DEHA = IF time>T_lag then 1 - exp(-(time - T_lag)^beta/alpha) else 0
beta = 1;dissolution shape
alpha = 1 ;dissolution half life
T_lag = 0.1 ; dissolution onset

Peff_human_DEHA = 4.885 ;10^(0.4926 * log10(Pcaco2_DEHA)-0.1454) ; 10^-4 cm/s
Peff_rat_DEHA = (Peff_human_DEHA - 0.03)/3.6 * 60 / 10000    ; cm/min, fitted
ka_du = 2 * 1.7 * Peff_rat_DEHA * 0.49/radius_intestine
ka_je = 2 * 1.7 * Peff_rat_DEHA * 1/radius_intestine
ka_il = 2 * 1.7 * Peff_rat_DEHA * 0.58/radius_intestine
ka_co = 2 * 1.7 * Peff_rat_DEHA * 0.03/radius_intestine

INIT X_st_DEHA = dose_PO_DEHA
INIT X_du_DEHA = 0
INIT X_je_DEHA = 0
INIT X_il_DEHA = 0
INIT X_co_DEHA = 0
INIT X_fe_DEHA = 0

d/dt(X_st_DEHA) = - k_st * X_st_DEHA
```

d/dt(X_du_DEHA) = k_st * X_st_DEHA - Frac_dis_DEHA * ka_du * X_du_DEHA - k_du * X_du_DEHA

d/dt(X_je_DEHA) = k_du * X_du_DEHA - Frac_dis_DEHA * ka_je * X_je_DEHA - k_je * X_je_DEHA

d/dt(X_il_DEHA) = k_je * X_je_DEHA - Frac_dis_DEHA * ka_il * X_il_DEHA - k_il * X_il_DEHA

d/dt(X_co_DEHA) = k_il * X_il_DEHA - Frac_dis_DEHA * ka_co * X_co_DEHA - k_co * X_co_DEHA

d/dt(X_fe_DEHA) = k_co * X_co_DEHA

k_st = 1.44 / 60 ;1/min

k_du = 2.46 / 60 ;1/min

k_je = 0.82 / 60 ;1/min

k_il = 0.82 / 60 ;1/min

k_co = 0.16 / 60 ;1/min

Xin_oral_g_DEHA = PO_scaler_DEHA * (Frac_dis_DEHA * ka_du * X_du_DEHA + Frac_dis_DEHA * ka_je * X_je_DEHA + Frac_dis_DEHA * ka_il * X_il_DEHA + Frac_dis_DEHA * ka_co * X_co_DEHA)

{distribution}

fup_DEHA_SPME = 0.0208

fup_DEHA = fup_DEHA_SPME

RB_DEHA = 1- Hct

EP_DEHA = 1 + (RB_DEHA-1)/Hct

Vss_DEHA = EP_DEHA * (Vve+Var) * Hct + (Vve+Var) * (1-Hct) + Kp_a_DEHA * Va + Kp_bo_DEHA * Vbo + Kp_b_DEHA * Vb + Kp_g_DEHA * Vg + Kp_h_DEHA * Vh + Kp_k_DEHA * Vk + Kp_li_DEHA * Vli + Kp_lg_DEHA * Vlg + Kp_m_DEHA * Vm + Kp_sk_DEHA * Vsk + Kp_sp_DEHA * Vsp

;Kp;

D = 4

fut_a_DEHA_homogenate = 0.02494

fut_b_DEHA_homogenate = 0.01704

fut_g_DEHA_homogenate = 0.1644

fut_h_DEHA_homogenate = 0.01668

fut_k_DEHA_homogenate = 0.03199

fut_li_DEHA_homogenate = 0.02036

fut_lg_DEHA_homogenate = 0.01063

fut_m_DEHA_homogenate = 0.01601

fut_sp_DEHA_homogenate = 0.01172

fut_a_DEHA = (1/D) / (1/fut_a_DEHA_homogenate - 1 + 1/D)

fut_b_DEHA = (1/D) / (1/fut_b_DEHA_homogenate - 1 + 1/D)

fut_g_DEHA = (1/D) / (1/fut_g_DEHA_homogenate - 1 + 1/D)

fut_h_DEHA = (1/D) / (1/fut_h_DEHA_homogenate - 1 + 1/D)

fut_k_DEHA = (1/D) / (1/fut_k_DEHA_homogenate - 1 + 1/D)

fut_li_DEHA = (1/D) / (1/fut_li_DEHA_homogenate - 1 + 1/D)

fut_lg_DEHA = (1/D) / (1/fut_lg_DEHA_homogenate - 1 + 1/D)

fut_m_DEHA = (1/D) / (1/fut_m_DEHA_homogenate - 1 + 1/D)

fut_sp_DEHA = (1/D) / (1/fut_sp_DEHA_homogenate - 1 + 1/D)

Kp_uu_DEHA = 1

Kp_a_DEHA = fup_DEHA / fut_a_DEHA * Kp_scaler_DEHA * Kp_uu_DEHA

Kp_b_DEHA = fup_DEHA / fut_b_DEHA * Kp_scaler_DEHA * Kp_uu_DEHA

Kp_g_DEHA = fup_DEHA / fut_g_DEHA * Kp_scaler_DEHA * Kp_uu_DEHA

Kp_h_DEHA = fup_DEHA / fut_h_DEHA * Kp_scaler_DEHA * Kp_uu_DEHA

Kp_k_DEHA = fup_DEHA / fut_k_DEHA * Kp_scaler_DEHA * Kp_uu_DEHA

Kp_li_DEHA = fup_DEHA / fut_li_DEHA * Kp_scaler_DEHA * Kp_uu_DEHA

Kp_lg_DEHA = fup_DEHA / fut_lg_DEHA * Kp_scaler_DEHA * Kp_uu_DEHA

Kp_m_DEHA = fup_DEHA / fut_m_DEHA * Kp_scaler_DEHA * Kp_uu_DEHA

Kp_sp_DEHA = fup_DEHA / fut_sp_DEHA * Kp_scaler_DEHA * Kp_uu_DEHA

;predicted by Berezhkovskiy;

Kp_bo_DEHA = 6.508 ;* Kp_scaler_DEHA

Kp_sk_DEHA = 22.79 ;* Kp_scaler_DEHA

; Permeability (cm/s) ;

;; PS (ml/min) ;;

PS_a_DEHA = P_PAMPA_DEHA * Seff_a * 60 * 10000

```
PS_bo_DEHA = P_PAMPA_DEHA * Seff_bo * 60 * 10000
PS_b_DEHA = P_PAMPA_DEHA * Seff_b * 60 * 10000
PS_g_DEHA = P_PAMPA_DEHA * Seff_g * 60 * 10000
PS_h_DEHA = P_PAMPA_DEHA * Seff_h * 60 * 10000
PS_k_DEHA = P_PAMPA_DEHA * Seff_k * 60 * 10000
PS_li_DEHA = P_PAMPA_DEHA * Seff_li * 60 * 10000
PS_lg_DEHA = P_PAMPA_DEHA * Seff_lg * 60 * 10000
PS_m_DEHA = P_PAMPA_DEHA * Seff_m * 60 * 10000
PS_sk_DEHA = P_PAMPA_DEHA * Seff_sk * 60 * 10000
PS_sp_DEHA = P_PAMPA_DEHA * Seff_sp * 60 * 10000

;; fd ;;
fd_a_DEHA = 1 - exp(-fup_DEHA * PS_a_DEHA/RB_DEHA/Q_a)
fd_bo_DEHA = 1 - exp(-fup_DEHA * PS_bo_DEHA /RB_DEHA/Q_bo)
fd_b_DEHA = 1 - exp(-fup_DEHA * PS_b_DEHA /RB_DEHA/Q_b)
fd_g_DEHA = 1 - exp(-fup_DEHA * PS_g_DEHA /RB_DEHA/Q_g)
fd_h_DEHA = 1 - exp(-fup_DEHA * PS_h_DEHA /RB_DEHA/Q_h)
fd_k_DEHA = 1 - exp(-fup_DEHA * PS_k_DEHA /RB_DEHA/Q_k)
fd_li_DEHA = 1 - exp(-fup_DEHA * PS_li_DEHA /RB_DEHA/Q_li)
fd_lg_DEHA = 1 - exp(-fup_DEHA * PS_lg_DEHA /RB_DEHA/Q_lg)
fd_m_DEHA = 1 - exp(-fup_DEHA * PS_m_DEHA /RB_DEHA/Q_m)
fd_sk_DEHA = 1 - exp(-fup_DEHA * PS_sk_DEHA /RB_DEHA/Q_sk)
fd_sp_DEHA = 1 - exp(-fup_DEHA * PS_sp_DEHA /RB_DEHA/Q_sp)


{metabolism & excretion}
;liver;
fumic_DEHA = fup_DEHA_SPME
k_mic_DEHA = 0.08915 ;/min
V_mic_DEHA = 1/0.5 ;mL/mg protein
CL_mic_DEHA = k_mic_DEHA * V_mic_DEHA ;mL/min/mg protein
CLint_u_mic_DEHA = CL_mic_DEHA /fumic_DEHA * MPPGL_mic * W_li ; mL/min
Xmet_li_mic_DEHA = CLint_u_mic_DEHA * C_li_DEHA * fup_DEHA/Kp_li_DEHA ; ng/min
```

```
;tissues other than liver;
k_a_DEHA_homogenate = 0.02436
k_b_DEHA_homogenate = 0.07674
k_g_DEHA_homogenate = 0.1392
k_h_DEHA_homogenate = 0.03162
k_k_DEHA_homogenate = 0.09506
k_lg_DEHA_homogenate = 0.03799
k_m_DEHA_homogenate = 0.03216
k_sp_DEHA_homogenate = 0.06294

k_a_DEHA_u = k_a_DEHA_homogenate / fut_a_DEHA_homogenate * D
k_b_DEHA_u = k_b_DEHA_homogenate / fut_b_DEHA_homogenate * D
k_g_DEHA_u = k_g_DEHA_homogenate / fut_g_DEHA_homogenate * D
k_h_DEHA_u = k_h_DEHA_homogenate / fut_h_DEHA_homogenate * D
k_k_DEHA_u = k_k_DEHA_homogenate / fut_k_DEHA_homogenate * D
k_lg_DEHA_u = k_lg_DEHA_homogenate / fut_lg_DEHA_homogenate * D
k_m_DEHA_u = k_m_DEHA_homogenate / fut_m_DEHA_homogenate * D
k_sp_DEHA_u = k_sp_DEHA_homogenate / fut_sp_DEHA_homogenate * D

;plasma;
k_plasma_DEHA = 0.1217 ;/min

ftissue_scaler = 1
ftissue_met_a_DEHA = 0 * ftissue_scaler
ftissue_met_b_DEHA = 0 * ftissue_scaler
ftissue_met_g_DEHA = 0.312 * ftissue_scaler
ftissue_met_h_DEHA = 0.198 * ftissue_scaler
ftissue_met_k_DEHA = 0.702 * ftissue_scaler
ftissue_met_lg_DEHA = 0.769 * ftissue_scaler
ftissue_met_m_DEHA = 0.390 * ftissue_scaler
ftissue_met_sp_DEHA = 0.272 * ftissue_scaler

;lymph;
```

k_lymph_DEHA = 0.0122

;renal clearance;

CL_renal_DEHA = 0 ;ml/min

;Additional clearance (vascular);

CL_vas_scaler = 1

CL_add_ar_DEHA = CL_vas_scaler * (k_a_DEHA_u * (1-ftissue_met_a_DEHA) * Va + k_b_DEHA_u * (1-ftissue_met_b_DEHA) * Vb + k_g_DEHA_u * (1-ftissue_met_g_DEHA) * Vg + k_h_DEHA_u * (1-ftissue_met_h_DEHA) * Vh + k_k_DEHA_u * (1-ftissue_met_k_DEHA) * Vk + k_m_DEHA_u * (1-ftissue_met_m_DEHA) * Vm + k_sp_DEHA_u * (1-ftissue_met_sp_DEHA) * Vsp) * fup_DEHA ;ml/min

CL_add_ve_DEHA = CL_vas_scaler * k_lg_DEHA_u * (1-ftissue_met_lg_DEHA) * Vlg * fup_DEHA;ml/min

{Initial Values}

INIT C_ar_DEHA = 1e-8

INIT C_ve_DEHA = 1e-8

INIT C_a_DEHA = 0

INIT C_bo_DEHA = 0

INIT C_b_DEHA = 0

INIT C_g_DEHA = 1e-8

INIT C_h_DEHA = 0

INIT C_k_DEHA = 0

INIT C_li_DEHA = 0

INIT C_lg_DEHA = 0

INIT C_m_DEHA = 0

INIT C_sk_DEHA = 0

INIT C_sp_DEHA = 0

INIT C_lymph_DEHA = 0

{Mass in}

Xin_ar_DEHA = Xout_lg_DEHA + Xin_lg_DEHA * (1-fd_lg_DEHA)/(fd_lg_DEHA)

Xin_ve_DEHA = IV_input_DEHA + Xout_a_DEHA + Xin_a_DEHA * (1-fd_a_DEHA)/(fd_a_DEHA) + Xout_bo_DEHA + Xin_bo_DEHA * (1-fd_bo_DEHA)/(fd_bo_DEHA) + Xout_b_DEHA + Xin_b_DEHA * (1-fd_b_DEHA)/(fd_b_DEHA) + Xout_h_DEHA + Xin_h_DEHA * (1-fd_h_DEHA)/(fd_h_DEHA) + Xout_k_DEHA + Xin_k_DEHA * (1-fd_k_DEHA)/(fd_k_DEHA) + Xout_li_DEHA + Xin_li_DEHA * (1-fd_li_DEHA)/(fd_li_DEHA) + Xout_m_DEHA + Xin_m_DEHA * (1-fd_m_DEHA)/(fd_m_DEHA) +

Xout_sk_DEHA + Xin_sk_DEHA * (1-fd_sk_DEHA)/(fd_sk_DEHA) + Q_rest * C_ar_DEHA * RB_DEHA + delay(Xout_lymph_DEHA,T_lag_lymph)

Xin_a_DEHA = fd_a_DEHA * Q_a * C_ar_DEHA * RB_DEHA

Xin_bo_DEHA = fd_bo_DEHA * Q_bo * C_ar_DEHA * RB_DEHA

Xin_b_DEHA = fd_b_DEHA * Q_b * C_ar_DEHA * RB_DEHA

Xin_g_DEHA = fd_g_DEHA * Q_g * C_ar_DEHA * RB_DEHA

Xin_h_DEHA = fd_h_DEHA * Q_h * C_ar_DEHA * RB_DEHA

Xin_k_DEHA = fd_k_DEHA * Q_k * C_ar_DEHA * RB_DEHA

Xin_li_DEHA = fd_li_DEHA * ((Q_li-Q_g-Q_sp) * C_ar_DEHA * RB_DEHA + fd_g_DEHA * Q_g * C_g_DEHA/Kp_g_DEHA*RB_DEHA + Xin_g_DEHA * (1-fd_g_DEHA)/(fd_g_DEHA) + Xout_sp_DEHA + Xin_sp_DEHA * (1-fd_sp_DEHA)/(fd_sp_DEHA))

Xin_lg_DEHA = fd_lg_DEHA * Q_lg * C_ve_DEHA * RB_DEHA

Xin_m_DEHA = fd_m_DEHA * Q_m * C_ar_DEHA * RB_DEHA

Xin_sk_DEHA = fd_sk_DEHA * Q_sk * C_ar_DEHA * RB_DEHA

Xin_sp_DEHA = fd_sp_DEHA * Q_sp * C_ar_DEHA * RB_DEHA

Xin_lymph_DEHA = CL_lymph * C_g_DEHA

{Mass out}

Xout_ar_DEHA = Q_lg * C_ar_DEHA * RB_DEHA

Xout_ve_DEHA = Q_lg * C_ve_DEHA * RB_DEHA

Xout_a_DEHA = fd_a_DEHA * Q_a * C_a_DEHA / Kp_a_DEHA * RB_DEHA

Xout_bo_DEHA = fd_bo_DEHA * Q_bo * C_bo_DEHA / Kp_bo_DEHA * RB_DEHA

Xout_b_DEHA = fd_b_DEHA * Q_b * C_b_DEHA / Kp_b_DEHA * RB_DEHA

Xout_g_DEHA = fd_g_DEHA * Q_g * C_g_DEHA / Kp_g_DEHA * RB_DEHA + CL_lymph * C_g_DEHA

Xout_h_DEHA = fd_h_DEHA * Q_h * C_h_DEHA / Kp_h_DEHA * RB_DEHA

Xout_k_DEHA = fd_k_DEHA * Q_k * C_k_DEHA / Kp_k_DEHA * RB_DEHA

Xout_li_DEHA = fd_li_DEHA * Q_li * C_li_DEHA / Kp_li_DEHA * RB_DEHA

Xout_lg_DEHA = fd_lg_DEHA * Q_lg * C_lg_DEHA / Kp_lg_DEHA * RB_DEHA

Xout_m_DEHA = fd_m_DEHA * Q_m * C_m_DEHA / Kp_m_DEHA * RB_DEHA

Xout_sk_DEHA = fd_sk_DEHA * Q_sk * C_sk_DEHA / Kp_sk_DEHA * RB_DEHA

Xout_sp_DEHA = fd_sp_DEHA * Q_sp * C_sp_DEHA / Kp_sp_DEHA * RB_DEHA

Xout_lymph_DEHA = (Q_lymph / Vlymph) * (C_lymph_DEHA * Vlymph) ;

{renal clearance}

Xrenal_DEHA = CL_renal_DEHA * C_ar_DEHA

{Metabolism}

Metabolism_scaler_DEHA = 1

Xmet_ar_DEHA = (k_plasma_DEHA * C_ar_DEHA * Var * (1-Hct) + CL_add_ar_DEHA * C_ar_DEHA) * Metabolism_scaler_DEHA

Xmet_ve_DEHA = (k_plasma_DEHA * C_ve_DEHA * Vve * (1-Hct) + CL_add_ve_DEHA * C_ve_DEHA) * Metabolism_scaler_DEHA ;+ Xmet_lg_cap_DEHA + Xmet_a_cap_DEHA_ve + Xmet_b_cap_DEHA_ve + Xmet_g_cap_DEHA_ve + Xmet_h_cap_DEHA_ve + Xmet_k_cap_DEHA_ve + Xmet_m_cap_DEHA_ve + Xmet_sp_cap_DEHA_ve

ratio = (k_plasma_DEHA * Var * (1-Hct) + CL_add_ar_DEHA) /(k_plasma_DEHA * Vve * (1-Hct) + CL_add_ve_DEHA)

Xmet_li_DEHA = Xmet_li_mic_DEHA * Metabolism_scaler_DEHA; ng/min

Xmet_a_DEHA = k_a_DEHA_u * ftissue_met_a_DEHA * C_a_DEHA * fut_a_DEHA * Va * Metabolism_scaler_DEHA

Xmet_b_DEHA = k_b_DEHA_u * ftissue_met_b_DEHA * C_b_DEHA * fut_b_DEHA * Vb * Metabolism_scaler_DEHA

Xmet_g_DEHA = k_g_DEHA_u * ftissue_met_g_DEHA * C_g_DEHA * fut_g_DEHA * Vg * Metabolism_scaler_DEHA

Xmet_h_DEHA = k_h_DEHA_u * ftissue_met_h_DEHA * C_h_DEHA * fut_h_DEHA * Vh * Metabolism_scaler_DEHA

Xmet_k_DEHA = k_k_DEHA_u * ftissue_met_k_DEHA * C_k_DEHA * fut_k_DEHA * Vk * Metabolism_scaler_DEHA

Xmet_lg_DEHA = k_lg_DEHA_u * ftissue_met_lg_DEHA * C_lg_DEHA * fut_lg_DEHA * Vlg * Metabolism_scaler_DEHA

Xmet_m_DEHA = k_m_DEHA_u * ftissue_met_m_DEHA * C_m_DEHA * fut_m_DEHA * Vm * Metabolism_scaler_DEHA

Xmet_sp_DEHA = k_sp_DEHA_u * ftissue_met_sp_DEHA * C_sp_DEHA * fut_sp_DEHA * Vsp * Metabolism_scaler_DEHA

Xmet_lymph_DEHA = Km_lymph_DEHA * k_lymph_DEHA/(Km_lymph_DEHA + C_lymph_DEHA) * C_lymph_DEHA * Vlymph * Metabolism_scaler_DEHA

{Differential Equations}

d/dt(C_ar_DEHA) = (1/Var/RB_DEHA) * (Xin_ar_DEHA - Xout_ar_DEHA - Xmet_ar_DEHA- Xrenal_DEHA)

d/dt(C_ve_DEHA) = (1/Vve/RB_DEHA) * (Xin_ve_DEHA - Xout_ve_DEHA - Xmet_ve_DEHA)

d/dt(C_a_DEHA) = (1/Va) * (Xin_a_DEHA - Xout_a_DEHA - Xmet_a_DEHA)

d/dt(C_bo_DEHA) = (1/Vbo) * (Xin_bo_DEHA - Xout_bo_DEHA)

d/dt(C_b_DEHA) = (1/Vb) * (Xin_b_DEHA - Xout_b_DEHA - Xmet_b_DEHA)

d/dt(C_g_DEHA) = (1/Vg) * (Xin_g_DEHA + Xin_oral_g_DEHA - Xout_g_DEHA - Xmet_g_DEHA)

d/dt(C_h_DEHA) = (1/Vh) * (Xin_h_DEHA - Xout_h_DEHA - Xmet_h_DEHA)

d/dt(C_k_DEHA) = (1/Vk) * (Xin_k_DEHA - Xout_k_DEHA - Xmet_k_DEHA)

d/dt(C_li_DEHA) = (1/Vli) * (Xin_li_DEHA - Xout_li_DEHA - Xmet_li_DEHA)

d/dt(C_lg_DEHA) = (1/Vlg) * (Xin_lg_DEHA - Xout_lg_DEHA - Xmet_lg_DEHA)

d/dt(C_m_DEHA) = (1/Vm) * (Xin_m_DEHA - Xout_m_DEHA - Xmet_m_DEHA)

d/dt(C_sk_DEHA) = (1/Vsk) * (Xin_sk_DEHA - Xout_sk_DEHA)

d/dt(C_sp_DEHA) = (1/Vsp) * (Xin_sp_DEHA - Xout_sp_DEHA - Xmet_sp_DEHA)

d/dt(C_lymph_DEHA) = (1/Vlymph) * (Xin_lymph_DEHA - Xout_lymph_DEHA - Xmet_lymph_DEHA)

{Unit conversion}

C_ar_DEHA_uM = C_ar_DEHA / MW_DEHA

C_ve_DEHA_uM = C_ve_DEHA / MW_DEHA

C_a_DEHA_uM = C_a_DEHA / MW_DEHA

C_bo_DEHA_uM = C_bo_DEHA / MW_DEHA

C_b_DEHA_uM = C_b_DEHA / MW_DEHA

C_g_DEHA_uM = C_g_DEHA / MW_DEHA

C_h_DEHA_uM = C_h_DEHA / MW_DEHA

C_k_DEHA_uM = C_k_DEHA / MW_DEHA

C_li_DEHA_uM = C_li_DEHA / MW_DEHA

C_lg_DEHA_uM = C_lg_DEHA / MW_DEHA

C_m_DEHA_uM = C_m_DEHA / MW_DEHA

C_sk_DEHA_uM = C_sk_DEHA / MW_DEHA

C_sp_DEHA_uM = C_sp_DEHA / MW_DEHA

{Compound physical & chemical properties}

MW_DEHA = 370.6 ;g/mol

{AUC}

INIT AUC_DEHA = 0

d/dt(AUC_DEHA) = C_ar_DEHA

```
INIT AUMC_DEHA = 0
d/dt(AUMC_DEHA) = t * C_ar_DEHA

INIT AUC_ve_DEHA = 0
d/dt(AUC_ve_DEHA) = C_ve_DEHA

;;;;;;;;;;;;;;;;;;;;;;;;;;
;;;;;;;;;;;MEHA;;;;;;;;;;;
;;;;;;;;;;;;;;;;;;;;;;;;;;

{administration}
dosing_time_IV = 5/60 ;min
IV_input_MEHA = IF t< dosing_time_IV THEN dose_IV_MEHA/dosing_time_IV ELSE 0
{distribution}

fup_MEHA_SPME = 0.0278
fup_MEHA = fup_MEHA_SPME

RB_MEHA = 0.592
EP_MEHA = 1 + (RB_MEHA-1)/Hct

;Kp;

D = 4

fut_a_MEHA_homogenate = 0.4306
fut_b_MEHA_homogenate = 0.04432
fut_g_MEHA_homogenate = 0.1348
fut_h_MEHA_homogenate = 0.3546
fut_k_MEHA_homogenate = 0.6838
fut_li_MEHA_homogenate = 0.5212
fut_lg_MEHA_homogenate = 0.1305
fut_m_MEHA_homogenate = 0.4458
fut_sp_MEHA_homogenate = 0.2684
```

```
fut_a_MEHA = (1/D) / (1/fut_a_MEHA_homogenate - 1 + 1/D)
fut_b_MEHA = (1/D) / (1/fut_b_MEHA_homogenate - 1 + 1/D)
fut_g_MEHA = (1/D) / (1/fut_g_MEHA_homogenate - 1 + 1/D)
fut_h_MEHA = (1/D) / (1/fut_h_MEHA_homogenate - 1 + 1/D)
fut_k_MEHA = (1/D) / (1/fut_k_MEHA_homogenate - 1 + 1/D)
fut_li_MEHA = (1/D) / (1/fut_li_MEHA_homogenate - 1 + 1/D)
fut_lg_MEHA = (1/D) / (1/fut_lg_MEHA_homogenate - 1 + 1/D)
fut_m_MEHA = (1/D) / (1/fut_m_MEHA_homogenate - 1 + 1/D)
fut_sp_MEHA = (1/D) / (1/fut_sp_MEHA_homogenate - 1 + 1/D)


Kp_scaler_MEHA = 1
Kp_uu_MEHA = 1
Kp_a_MEHA = fup_MEHA / fut_a_MEHA * Kp_scaler_MEHA * Kp_uu_MEHA
Kp_b_MEHA = fup_MEHA / fut_b_MEHA * Kp_scaler_MEHA * Kp_uu_MEHA
Kp_g_MEHA = fup_MEHA / fut_g_MEHA * Kp_scaler_MEHA * Kp_uu_MEHA
Kp_h_MEHA = fup_MEHA / fut_h_MEHA * Kp_scaler_MEHA * Kp_uu_MEHA
Kp_k_MEHA = fup_MEHA / fut_k_MEHA * Kp_scaler_MEHA * Kp_uu_MEHA
Kp_li_MEHA = fup_MEHA / fut_li_MEHA * Kp_scaler_MEHA * Kp_uu_MEHA
Kp_lg_MEHA = fup_MEHA / fut_lg_MEHA * Kp_scaler_MEHA * Kp_uu_MEHA
Kp_m_MEHA = fup_MEHA / fut_m_MEHA * Kp_scaler_MEHA * Kp_uu_MEHA
Kp_sp_MEHA = fup_MEHA / fut_sp_MEHA * Kp_scaler_MEHA * Kp_uu_MEHA


;predicted by Schmitt;
Kp_bo_MEHA = 0.6388 ;1.904360682;
Kp_sk_MEHA = 3.276 ;1.497723859;

; Permeability (cm/s) ;
P_PAMPA_MEHA = 4.88e-5

;; PS (ml/min) ;;
PS_a_MEHA = P_PAMPA_MEHA * Seff_a * 60 * 10000
PS_bo_MEHA = P_PAMPA_MEHA * Seff_bo * 60 * 10000
PS_b_MEHA = P_PAMPA_MEHA * Seff_b * 60 * 10000
```

```
PS_g_MEHA = P_PAMPA_MEHA * Seff_g * 60 * 10000
PS_h_MEHA = P_PAMPA_MEHA * Seff_h * 60 * 10000
PS_k_MEHA = P_PAMPA_MEHA * Seff_k * 60 * 10000
PS_li_MEHA = P_PAMPA_MEHA * Seff_li * 60 * 10000
PS_lg_MEHA = P_PAMPA_MEHA * Seff_lg * 60 * 10000
PS_m_MEHA = P_PAMPA_MEHA * Seff_m * 60 * 10000
PS_sk_MEHA = P_PAMPA_MEHA * Seff_sk * 60 * 10000
PS_sp_MEHA = P_PAMPA_MEHA * Seff_sp * 60 * 10000

;; fd ;;
fd_a_MEHA = 1 - exp(-fup_MEHA * PS_a_MEHA/RB_MEHA/Q_a)
fd_bo_MEHA = 1 - exp(-fup_MEHA * PS_bo_MEHA /RB_MEHA/Q_bo)
fd_b_MEHA = 1 - exp(-fup_MEHA * PS_b_MEHA /RB_MEHA/Q_b)
fd_g_MEHA = 1 - exp(-fup_MEHA * PS_g_MEHA /RB_MEHA/Q_g)
fd_h_MEHA = 1 - exp(-fup_MEHA * PS_h_MEHA /RB_MEHA/Q_h)
fd_k_MEHA = 1 - exp(-fup_MEHA * PS_k_MEHA /RB_MEHA/Q_k)
fd_li_MEHA = 1 - exp(-fup_MEHA * PS_li_MEHA /RB_MEHA/Q_li)
fd_lg_MEHA = 1 - exp(-fup_MEHA * PS_lg_MEHA /RB_MEHA/Q_lg)
fd_m_MEHA = 1 - exp(-fup_MEHA * PS_m_MEHA /RB_MEHA/Q_m)
fd_sk_MEHA = 1 - exp(-fup_MEHA * PS_sk_MEHA /RB_MEHA/Q_sk)
fd_sp_MEHA = 1 - exp(-fup_MEHA * PS_sp_MEHA /RB_MEHA/Q_sp)

{metabolism & excretion}
;liver;

fumic_MEHA = fup_MEHA
k_mic_MEHA =    0.8113 * MEHA_k_scaler ;/min
V_mic_MEHA = 1/0.5 ;mL/mg protein
CL_mic_MEHA = k_mic_MEHA * V_mic_MEHA ;mL/min/mg protein
CLint_u_mic_MEHA = CL_mic_MEHA /fumic_MEHA * MPPGL_mic * W_li ; mL/min
Xmet_li_mic_MEHA = CLint_u_mic_MEHA * C_li_MEHA * fup_MEHA/Kp_li_MEHA ; ng/min

;tissues;
D = 4
MEHA_k_scaler = 1
```

```
k_a_MEHA_homogenate = 2.009
k_b_MEHA_homogenate = 0.05720
k_g_MEHA_homogenate = 8.066
k_h_MEHA_homogenate = 0.5530
k_k_MEHA_homogenate = 4.580
k_lg_MEHA_homogenate = 2.011
k_m_MEHA_homogenate = 0.2287
k_sp_MEHA_homogenate = 0.2748
k_lymph_MEHA = 0.146

k_a_MEHA_u = k_a_MEHA_homogenate / fut_a_MEHA_homogenate * D
k_b_MEHA_u = k_b_MEHA_homogenate / fut_b_MEHA_homogenate * D
k_g_MEHA_u = k_g_MEHA_homogenate / fut_g_MEHA_homogenate * D
k_h_MEHA_u = k_h_MEHA_homogenate / fut_h_MEHA_homogenate * D
k_k_MEHA_u = k_k_MEHA_homogenate / fut_k_MEHA_homogenate * D
k_lg_MEHA_u = k_lg_MEHA_homogenate / fut_lg_MEHA_homogenate * D
k_m_MEHA_u = k_m_MEHA_homogenate / fut_m_MEHA_homogenate * D
k_sp_MEHA_u = k_sp_MEHA_homogenate / fut_sp_MEHA_homogenate * D

k_a_MEHA = k_a_MEHA_u * fut_a_MEHA * MEHA_k_scaler
k_b_MEHA = k_b_MEHA_u * fut_b_MEHA * MEHA_k_scaler
k_g_MEHA = k_g_MEHA_u * fut_g_MEHA * MEHA_k_scaler
k_h_MEHA = k_h_MEHA_u * fut_h_MEHA * MEHA_k_scaler
k_k_MEHA = k_k_MEHA_u * fut_k_MEHA * MEHA_k_scaler
k_lg_MEHA = k_lg_MEHA_u * fut_lg_MEHA * MEHA_k_scaler
k_m_MEHA = k_m_MEHA_u * fut_m_MEHA * MEHA_k_scaler
k_sp_MEHA = k_sp_MEHA_u * fut_sp_MEHA * MEHA_k_scaler

;plasma;
k_plasma_MEHA = 0.03138 ;/min

;renal clearance;
CL_renal_MEHA = 0 ;ml/min
```

;Additional clearance;

CL_add_MEHA =0 ;ml/min

{Initial Values}

INIT C_ar_MEHA = 1e-8

INIT C_ve_MEHA = 1e-8

INIT C_a_MEHA = 0

INIT C_bo_MEHA = 0

INIT C_b_MEHA = 0

INIT C_g_MEHA = 0

INIT C_h_MEHA = 0

INIT C_k_MEHA = 0

INIT C_li_MEHA = 0

INIT C_lg_MEHA = 0

INIT C_m_MEHA = 0

INIT C_sk_MEHA = 0

INIT C_sp_MEHA = 0

INIT C_lymph_MEHA = 0

C_lymph_MEHA_ss = IF dose_PO_DEHA > 0 THEN 336 ELSE 0

{Mass in}

Xin_ar_MEHA = Xout_lg_MEHA + Xin_lg_MEHA * (1-fd_lg_MEHA)/(fd_lg_MEHA) + Xmet_ar_DEHA * MW_MEHA/MW_DEHA

Xin_ve_MEHA = IV_input_MEHA + Xout_a_MEHA + Xin_a_MEHA * (1-fd_a_MEHA)/(fd_a_MEHA) + Xout_bo_MEHA + Xin_bo_MEHA * (1-fd_bo_MEHA)/(fd_bo_MEHA) + Xout_b_MEHA + Xin_b_MEHA * (1-fd_b_MEHA)/(fd_b_MEHA) + Xout_h_MEHA + Xin_h_MEHA * (1-fd_h_MEHA)/(fd_h_MEHA) + Xout_k_MEHA + Xin_k_MEHA * (1-fd_k_MEHA)/(fd_k_MEHA) + Xout_li_MEHA + Xin_li_MEHA * (1-fd_li_MEHA)/(fd_li_MEHA) + Xout_m_MEHA + Xin_m_MEHA * (1-fd_m_MEHA)/(fd_m_MEHA) + Xout_sk_MEHA + Xin_sk_MEHA * (1-fd_sk_MEHA)/(fd_sk_MEHA) + Q_rest * C_ar_MEHA * RB_MEHA + Xmet_ve_DEHA * MW_MEHA/MW_DEHA + Xout_lymph_MEHA

Xin_a_MEHA = fd_a_MEHA * Q_a * C_ar_MEHA * RB_MEHA + Xmet_a_DEHA * MW_MEHA/MW_DEHA

Xin_bo_MEHA = fd_bo_MEHA * Q_bo * C_ar_MEHA * RB_MEHA

Xin_b_MEHA = fd_b_MEHA * Q_b * C_ar_MEHA * RB_MEHA + Xmet_b_DEHA * MW_MEHA/MW_DEHA

Xin_g_MEHA = fd_g_MEHA * Q_g * C_ar_MEHA * RB_MEHA + Xmet_g_DEHA * MW_MEHA/MW_DEHA

Xin_h_MEHA = fd_h_MEHA * Q_h * C_ar_MEHA * RB_MEHA + Xmet_h_DEHA * MW_MEHA/MW_DEHA

Xin_k_MEHA = fd_k_MEHA * Q_k * C_ar_MEHA * RB_MEHA + Xmet_k_DEHA * MW_MEHA/MW_DEHA

Xin_li_MEHA = fd_li_MEHA * ((Q_li-Q_g-Q_sp) * C_ar_MEHA * RB_MEHA + fd_g_MEHA * Q_g * C_g_MEHA / Kp_g_MEHA * RB_MEHA + Xin_g_MEHA * (1-fd_g_MEHA)/(fd_g_MEHA) + Xout_sp_MEHA + Xin_sp_MEHA * (1-fd_sp_MEHA)/(fd_sp_MEHA)) + Xmet_li_DEHA * MW_MEHA/MW_DEHA

Xin_lg_MEHA = fd_lg_MEHA * Q_lg * C_ve_MEHA * RB_MEHA + Xmet_lg_DEHA * MW_MEHA/MW_DEHA

Xin_m_MEHA = fd_m_MEHA * Q_m * C_ar_MEHA * RB_MEHA + Xmet_m_DEHA * MW_MEHA/MW_DEHA

Xin_sk_MEHA = fd_sk_MEHA * Q_sk * C_ar_MEHA * RB_MEHA

Xin_sp_MEHA = fd_sp_MEHA * Q_sp * C_ar_MEHA * RB_MEHA + Xmet_sp_DEHA * MW_MEHA/MW_DEHA

Xin_lymph_MEHA = Xmet_lymph_DEHA * MW_MEHA / MW_DEHA

{Mass out}

Xout_ar_MEHA = Q_lg * C_ar_MEHA * RB_MEHA

Xout_ve_MEHA = Q_lg * C_ve_MEHA * RB_MEHA

Xout_a_MEHA = fd_a_MEHA * Q_a * C_a_MEHA / Kp_a_MEHA * RB_MEHA

Xout_bo_MEHA = fd_bo_MEHA * Q_bo * C_bo_MEHA / Kp_bo_MEHA * RB_MEHA

Xout_b_MEHA = fd_b_MEHA * Q_b * C_b_MEHA / Kp_b_MEHA * RB_MEHA

Xout_g_MEHA = fd_g_MEHA * Q_g * C_g_MEHA / Kp_g_MEHA * RB_MEHA

Xout_h_MEHA = fd_h_MEHA * Q_h * C_h_MEHA / Kp_h_MEHA * RB_MEHA

Xout_k_MEHA = fd_k_MEHA * Q_k * C_k_MEHA / Kp_k_MEHA * RB_MEHA

Xout_li_MEHA = fd_li_MEHA * Q_li * C_li_MEHA / Kp_li_MEHA * RB_MEHA

Xout_lg_MEHA = fd_lg_MEHA * Q_lg * C_lg_MEHA / Kp_lg_MEHA * RB_MEHA

Xout_m_MEHA = fd_m_MEHA * Q_m * C_m_MEHA / Kp_m_MEHA * RB_MEHA

Xout_sk_MEHA = fd_sk_MEHA * Q_sk * C_sk_MEHA / Kp_sk_MEHA * RB_MEHA

Xout_sp_MEHA = fd_sp_MEHA * Q_sp * C_sp_MEHA / Kp_sp_MEHA * RB_MEHA

Xout_lymph_MEHA = Q_lymph * C_lymph_MEHA

{renal clearance}

Xrenal_MEHA = CL_renal_MEHA * C_ar_MEHA

{Additional clearance}

Xmet_add_MEHA = 0

{Metabolism}

Metabolism_scaler_MEHA = 1

Xmet_ar_MEHA = k_plasma_MEHA * C_ar_MEHA * Var * (1-Hct) * Metabolism_scaler_MEHA

Xmet_ve_MEHA = k_plasma_MEHA * C_ve_MEHA * Vve * (1-Hct) * Metabolism_scaler_MEHA

Xmet_li_MEHA = Xmet_li_mic_MEHA * Metabolism_scaler_MEHA

Xmet_a_MEHA = k_a_MEHA * C_a_MEHA * Va * Metabolism_scaler_MEHA

Xmet_b_MEHA = k_b_MEHA * C_b_MEHA * Vb * Metabolism_scaler_MEHA

Xmet_g_MEHA = k_g_MEHA * C_g_MEHA * Vg * Metabolism_scaler_MEHA

Xmet_h_MEHA = k_h_MEHA * C_h_MEHA * Vh * Metabolism_scaler_MEHA

Xmet_k_MEHA = k_k_MEHA * C_k_MEHA * Vk * Metabolism_scaler_MEHA

Xmet_lg_MEHA = k_lg_MEHA * C_lg_MEHA * Vlg * Metabolism_scaler_MEHA

Xmet_m_MEHA = k_m_MEHA * C_m_MEHA * Vm * Metabolism_scaler_MEHA

Xmet_sp_MEHA = k_sp_MEHA * C_sp_MEHA * Vsp * Metabolism_scaler_MEHA

Xmet_lymph_MEHA = k_lymph_MEHA * C_lymph_MEHA * Vlymph

Xmet_MEHA = Xmet_ar_MEHA + Xmet_ve_MEHA + Xmet_li_MEHA + Xmet_a_MEHA + Xmet_b_MEHA + Xmet_g_MEHA + Xmet_h_MEHA + Xmet_k_MEHA + Xmet_lg_MEHA + Xmet_m_MEHA + Xmet_sp_MEHA

CL_ar_MEHA = Xmet_ar_MEHA / C_ar_MEHA

CL_ve_MEHA = Xmet_ve_MEHA / C_ve_MEHA

CL_a_MEHA = Xmet_a_MEHA / C_ar_MEHA

CL_b_MEHA = Xmet_b_MEHA / C_ar_MEHA

CL_h_MEHA = Xmet_h_MEHA / C_ar_MEHA

CL_k_MEHA = Xmet_k_MEHA / C_ar_MEHA

CL_lg_MEHA = Xmet_lg_MEHA / C_ve_MEHA

CL_m_MEHA = Xmet_m_MEHA / C_ar_MEHA

CL_li_MEHA = Xmet_li_MEHA / C_ar_MEHA

CL_splanchnic_MEHA = (Xmet_li_MEHA + Xmet_g_MEHA + Xmet_sp_MEHA) / C_ar_MEHA

CL_sum_MEHA = CL_ar_MEHA + CL_ve_MEHA + CL_a_MEHA + CL_b_MEHA + CL_h_MEHA + CL_k_MEHA + CL_lg_MEHA + CL_m_MEHA + CL_splanchnic_MEHA

CL_sys_MEHA = Xmet_MEHA / C_ar_MEHA

{Differential Equations}

d/dt(C_ar_MEHA) = (1/Var/RB_MEHA) * (Xin_ar_MEHA - Xout_ar_MEHA - Xmet_ar_MEHA - Xmet_add_MEHA - Xrenal_MEHA)

```
d/dt(C_ve_MEHA) = (1/Vve/RB_MEHA) * (Xin_ve_MEHA - Xout_ve_MEHA - Xmet_ve_MEHA)
d/dt(C_a_MEHA) = (1/Va) * (Xin_a_MEHA - Xout_a_MEHA - Xmet_a_MEHA)
d/dt(C_bo_MEHA) = (1/Vbo) * (Xin_bo_MEHA - Xout_bo_MEHA)
d/dt(C_b_MEHA) = (1/Vb) * (Xin_b_MEHA - Xout_b_MEHA - Xmet_b_MEHA )
d/dt(C_g_MEHA) = (1/Vg) * (Xin_g_MEHA - Xout_g_MEHA - Xmet_g_MEHA)
d/dt(C_h_MEHA) = (1/Vh) * (Xin_h_MEHA - Xout_h_MEHA - Xmet_h_MEHA)
d/dt(C_k_MEHA) = (1/Vk) * (Xin_k_MEHA - Xout_k_MEHA - Xmet_k_MEHA)
d/dt(C_li_MEHA) = (1/Vli) * (Xin_li_MEHA - Xout_li_MEHA - Xmet_li_MEHA)
d/dt(C_lg_MEHA) = (1/Vlg) * (Xin_lg_MEHA - Xout_lg_MEHA - Xmet_lg_MEHA)
d/dt(C_m_MEHA) = (1/Vm) * (Xin_m_MEHA - Xout_m_MEHA - Xmet_m_MEHA)
d/dt(C_sk_MEHA) = (1/Vsk) * (Xin_sk_MEHA - Xout_sk_MEHA)
d/dt(C_sp_MEHA) = (1/Vsp) * (Xin_sp_MEHA - Xout_sp_MEHA - Xmet_sp_MEHA)
d/dt(C_lymph_MEHA) = (1/Vlymph) * (Xin_lymph_MEHA - Xout_lymph_MEHA - Xmet_lymph_MEHA)


{Unit conversion}
C_ar_MEHA_uM = C_ar_MEHA / MW_MEHA
C_ve_MEHA_uM = C_ve_MEHA / MW_MEHA
C_a_MEHA_uM = C_a_MEHA / MW_MEHA
C_bo_MEHA_uM = C_bo_MEHA / MW_MEHA
C_b_MEHA_uM = C_b_MEHA / MW_MEHA
C_g_MEHA_uM = C_g_MEHA / MW_MEHA
C_h_MEHA_uM = C_h_MEHA / MW_MEHA
C_k_MEHA_uM = C_k_MEHA / MW_MEHA
C_li_MEHA_uM = C_li_MEHA / MW_MEHA
C_lg_MEHA_uM = C_lg_MEHA / MW_MEHA
C_m_MEHA_uM = C_m_MEHA / MW_MEHA
C_sk_MEHA_uM = C_sk_MEHA / MW_MEHA
C_sp_MEHA_uM = C_sp_MEHA / MW_MEHA


{Compound physical & chemical properties}
MW_MEHA = 258.35 ;g/mol

{AUC}
```

```
INIT AUC_MEHA = 0
d/dt(AUC_MEHA) = C_ar_MEHA

INIT AUMC_MEHA = 0
d/dt(AUMC_MEHA) = t * C_ar_MEHA

INIT AUC_ve_MEHA = 0
d/dt(AUC_ve_MEHA) = C_ve_MEHA



;;;;;;;;;;;;;;;;;;;;;;;;;;;;;;;;;;;;;;
;;;;;Rat physiological parameters;;;;;
;;;;;;;;;;;;;;;;;;;;;;;;;;;;;;;;;;;;;;

{GI tract}

radius_intestine = 0.18 ; cm, Issa et al., 2003

{Hematocrit}

Hct = 7.2 / (10.04 + 7.2)

{tissue volume (mL) } ;SimCYP rat ver 22



Vve = (10.04 + 7.2) * 2/3 ; vein (blood)
Var = (10.04 + 7.2) * 1/3 ; artery (blood)
Va = 16.66 ; adipose
Vbo = 15.68 ; bone
Vb = 1.24 ; brain
Vg = 6.19 ; gut
Vh = 1.05 ; heart
Vk = 2.19 ; kidney
Vli = 8.57 ; liver
Vlg = 1.24 ; lung
```

Vm = 116.13 ; muscle

Vsk = 39.41 ; skin

Vsp = 0.57 ; spleen

Vrest = 250 - (Vve + Var + Va + Vbo + Vb + Vg + Vh + Vk + Vli + Vlg + Vm + Vsk + Vsp)

Vlymph = 17.24

{Blood flow (mL/min)} ;SimCYP rat ver 22

Q = 80 ; cardiac output

Q_lg = Q ; lung

Q_a = Q * 5.9/100 ; adipose

Q_bo = Q * 10.1/100 ; bone

Q_b = Q * 1.4/100 ; brain

Q_h = Q * 4/100 ; heart

Q_k = Q * 14.5/100 ; kidney

Q_li = Q * 24.2/100 ; liver

Q_sp = Q * 1.1/100 ; spleen

Q_g = Q * 10.1/100 ; gut

Q_m = Q * 23.7/100 ; muscle

Q_sk = Q * 5.1/100 ; skin

Q_rest = Q - (Q_a+Q_bo+Q_b+Q_h+Q_k+Q_li+Q_m+Q_sk); rest of body

Q_lymph = 2.5/60

{weight (g)}

BW = 250

W_a = Va * 0.9

W_bo = Vbo * 1.85

W_b = Vb * 1

W_h = Vh * 1

W_k = Vk * 1

W_li = Vli * 1

W_lg = Vlg * 1

W_sp = Vsp * 1

W_g = Vg * 1

W_m = Vm * 1

W_sk = Vsk * 1

{liver}

MPPGL_mic = 44.5 ; mg microsomal protein / g liver : https://doi.org/10.1002/bdd.1959

MPPGL_S9 = 115.2 ; mg S9 protein / g liver ; inhouse measured

{Seff (m^2)}

Seff_a = 3.02 * W_a

Seff_bo = 2.34 * W_bo

Seff_b = 1.67 * W_b

Seff_g = 16.1 * W_g

Seff_h = 18.1 * W_h

Seff_k = 37.4 * W_k

Seff_li = 14.8 * W_li

Seff_lg = 14.4 * W_lg

Seff_m = 5.71 * W_m

Seff_sk = 0.843 * W_sk

Seff_sp = 4.56 * W_sp

;;;;;;;;;;;;;;;;;;;;;;;;;;;;;

;;;;;;;;Abbreviation;;;;;;;;

;;;;;;;;;;;;;;;;;;;;;;;;;;;;;

; Volume (V)

; Blood flow (Q)

; Hematocrit (Hct)

; Weight (W)

; Vein (ve)

; Artery (ar)

; Adipose (a)

; Bone (bo)

; Brain (b)

; Gut (g)

; Heart (h)

; Kidneys (k)

; Liver (li)

; Lung (lg)

; Muscle (m)

; Plasma (p)

; Skin (sk)

; Spleen (sp)

; mg protein per gram liver (MPPGL)
